# Age effects on cognitive functions and speech-in-noise processing: An event-related potential study with cochlear-implant users and normal-hearing listeners

**DOI:** 10.3389/fnins.2022.1005859

**Published:** 2022-12-22

**Authors:** Pauline Burkhardt, Verena Müller, Hartmut Meister, Anna Weglage, Ruth Lang-Roth, Martin Walger, Pascale Sandmann

**Affiliations:** ^1^Department of Otorhinolaryngology, Head and Neck Surgery, Audiology and Pediatric Audiology, Cochlear Implant Center, Faculty of Medicine and University Hospital Cologne, University of Cologne, Cologne, Germany; ^2^Jean-Uhrmacher-Institute for Clinical ENT-Research, University of Cologne, Cologne, Germany

**Keywords:** cochlear implant, event-related potential, ERP, age, cognition, speech processing, speech-in-noise, N400

## Abstract

A cochlear implant (CI) can partially restore hearing in individuals with profound sensorineural hearing loss. However, electrical hearing with a CI is limited and highly variable. The current study aimed to better understand the different factors contributing to this variability by examining how age affects cognitive functions and cortical speech processing in CI users. Electroencephalography (EEG) was applied while two groups of CI users (young and elderly; *N* = 13 each) and normal-hearing (NH) listeners (young and elderly; *N* = 13 each) performed an auditory sentence categorization task, including semantically correct and incorrect sentences presented either with or without background noise. Event-related potentials (ERPs) representing earlier, sensory-driven processes (N1-P2 complex to sentence onset) and later, cognitive-linguistic integration processes (N400 to semantically correct/incorrect sentence-final words) were compared between the different groups and speech conditions. The results revealed reduced amplitudes and prolonged latencies of auditory ERPs in CI users compared to NH listeners, both at earlier (N1, P2) and later processing stages (N400 effect). In addition to this *hearing-group effect*, CI users and NH listeners showed a comparable *background-noise effect*, as indicated by reduced hit rates and reduced (P2) and delayed (N1/P2) ERPs in conditions with background noise. Moreover, we observed an *age effect* in CI users and NH listeners, with young individuals showing improved specific cognitive functions (working memory capacity, cognitive flexibility and verbal learning/retrieval), reduced latencies (N1/P2), decreased N1 amplitudes and an increased N400 effect when compared to the elderly. In sum, our findings extend previous research by showing that the CI users’ speech processing is impaired not only at earlier (sensory) but also at later (semantic integration) processing stages, both in conditions with and without background noise. Using objective ERP measures, our study provides further evidence of strong age effects on cortical speech processing, which can be observed in both the NH listeners and the CI users. We conclude that elderly individuals require more effortful processing at sensory stages of speech processing, which however seems to be at the cost of the limited resources available for the later semantic integration processes.

## Introduction

Cochlear implantation is a well-established procedure to treat patients with severe to profound sensorineural hearing loss. The cochlear implant (CI) is a partially implantable hearing system where an electrode array is surgically inserted into the cochlea to electrically stimulate the fibers of the auditory nerve. However, due to the CI’s limited temporal and spectral information, there are remarkable shortcomings in electrical hearing (Drennan and Rubinstein, 2008), and the central nervous system needs to adapt to this artificial sound. Nevertheless, many CI recipients show gradual improvement in their ability to recognize speech within the first 12 months after implantation (Lenarz et al., 2012). Importantly, these auditory improvements following implantation are not limited to young CI recipients but can also be observed in elderly and even in geriatric patients (Lenarz et al., 2012).

The speech recognition ability with a CI – also referred to as the CI outcome – is highly variable across the CI users (Lazard et al., 2012a). This variability can be (at least partially) accounted by inter-individual differences concerning the implant (Holden et al., 2013), the physiology of the auditory system (Nadol et al., 1989; Shepherd and Javel, 1997), the capacity for neuroplasticity in the auditory cortex (Sandmann et al., 2015), the lip-reading ability and cognitive skills, for instance verbal learning and working memory (Heydebrand et al., 2007). Moreover, the age of implantation can substantially affect the CI outcome particularly when the individuals become deaf *before* language acquisition. These so-called prelingually deafened individuals often show remarkable success in spoken language acquisition when they are fitted with a CI *early* but not late in childhood (Svirsky et al., 2000; McConkey Robbins et al., 2004; Kral and Sharma, 2012). Importantly, electrical stimulation in prelingually deafened, early implanted CI children allows a normal development of the central auditory system (Sharma et al., 2002).

Previous results with adult CI users who acquired deafness *after* language acquisition (i.e., postlingually deafened) have indicated poorer speech recognition ability in older compared to younger CI users, in particular in speech conditions with background noise (Lenarz et al., 2012). Age effects on speech-understanding abilities have also been reported in numerous studies with normal-hearing (NH) listeners (Humes and Dubno, 2010). The observed difficulties with speech-in-noise conditions in elderly individuals may be explained by age-related changes in the auditory periphery and in the central auditory system, leading to alterations in perceptual input and temporal response properties of cortical neurons (Tremblay et al., 2003; Martin and Jerger, 2005; Humes et al., 2012). Additional reasons for the difficulties experienced by elderly individuals are age-related structural declines in auditory- and cognition-related brain areas (Wong et al., 2010; Du et al., 2016; Giroud et al., 2021) as well as a decrement in cognitive functions, such as working memory (Salthouse, 1996; Pichora-Fuller and Singh, 2006; Zekveld et al., 2011).

In the clinical context, the CI outcome is typically assessed by means of speech audiometry, using words (Hahlbrock, 1953) or sentences presented either with or without background noise (Hochmair-Desoyer et al., 1997). The interpretation of these behavioral measures is, however, limited, as they reflect the output of multiple sensory and cognitive processes. By contrast, event-related potentials (ERPs) derived from continuous electroencephalography (EEG) provide a continuous measure of cortical speech processing with a high temporal resolution. Thus, ERPs allow the tracking of auditory signal propagation (Michel and Murray, 2012; Biasiucci et al., 2019) that is organized in a semi-hierarchical and highly parallel way (e.g., Kaas and Hackett, 2000). For instance, the auditory N1 and P2 ERPs (negative/positive potential around 100/200 ms after stimulus onset, respectively) reflect low-level sensory processing in a distributed cortical network, including the primary auditory cortex, the superior temporal cortices, and fronto-parietal structures (Näätänen and Picton, 1987; Bosnyak et al., 2004; Ahveninen et al., 2006). However, this sensory processing at N1 and P2 latency appears to be reduced and delayed in CI users when compared to NH listeners (Beynon et al., 2005; Sandmann et al., 2009; Henkin et al., 2014; Finke et al., 2016a) and it seems to be modulated by a number of factors, including age (Tremblay et al., 2003) and background noise (Billings et al., 2009; Finke et al., 2016a).

ERPs provide an interesting tool for studying not only low-level sensory but also higher cognitive processes required for language comprehension (for reviews see Friederici, 2006; Duncan et al., 2009). The N400 component, which is modulated by the semantic congruity and expectancy, is an important ERP marker in relation to sentence processing. According to current frameworks, the N400 reflects the amount of neural effort of semantic integration (“integration view”), or the facilitation of the lexical access due to contextual pre-activation (“lexical view”; Lau et al., 2008). For the present study it is important to take into account the results of earlier experiments which showed that the N400 response of NH listeners is reduced by age (Kutas and Iragui, 1998; Faustmann et al., 2007; Xu et al., 2017) and acoustic stimulus degradation (Aydelott et al., 2006; Obleser and Kotz, 2011). Although previous results suggest delayed N400 ERPs in CI users when compared to NH listeners (Hahne et al., 2012), it is currently unknown whether CI users show N400 modulation effects similar to NH listeners, especially in terms of age and (additional) auditory signal degradation, in particular background noise.

The current study intends to grasp a better understanding of the effects of age and background noise on cortical speech processing with a CI. Two hearing groups – CI users and NH listeners – across different age ranges (young/elderly) were tested with an auditory sentence categorization task (Hahne et al., 2012), using semantically correct and incorrect (i.e., violated) sentences that were presented either with or without background noise. We compared earlier, sensory-driven processes (reflected by the N1-P2 complex to sentence onset) and later, cognitive-linguistic integration processes (reflected by the N400 to the semantically correct/incorrect sentence-final word, also referred to as the “critical word”) between the different groups and speech conditions. Following the results of NH listeners (Kutas and Iragui, 1998; Tremblay et al., 2003; Billings et al., 2009), we expected that the CI users would show effects of age and background noise on cortical speech processing at the N1, P2, and N400 time ranges as well. Furthermore, we predicted that difficulties in the processing of the limited CI speech signal would result in a delay in CI users’ N1 and N400 ERP latencies when compared to NH listeners (Hahne et al., 2012; Finke et al., 2016b). Following the results from NH listeners tested with noise-vocoded speech stimuli (Rosemann et al., 2017) and hearing-impaired individuals (with moderate to severe hearing loss; Souza et al., 2015) we expected a relationship between working memory and speech-in-noise processing in young and elderly CI users.

## Materials and methods

### Participants

In total, fifty-five participants were included in the current study. Three of these participants had to be excluded from further analyses because of an extremely poor performance in speech audiometry (*N* = 1; Göttingen sentence test in noise > 10 dB signal-to-noise ratio) and due to massive muscular artifacts in the EEG (*N* = 2). Thus, 52 participants were included for further analyses, including 26 CI users and the same number of NH controls. There were two age groups (young/elderly) for each of the two hearing groups (CI users/NH listeners). This resulted in four subgroups of participants, each consisting of 13 subjects: young CI users (9 female, mean age and SD: 25.5 ± 4.9 years, range: 19–37 years), elderly CI users (9 female, mean age and SD: 71.1 ± 6.6 years, range: 60–79 years), young NH listeners (9 female, mean age and SD: 28.7 ± 5.0 years, range: 24–40 years), and elderly NH listeners (9 female, mean age and SD: 68.6 ± 6.0 years, range: 61–78 years).

Detailed information about the implant systems and the demographic variables of the young and elderly CI users are summarized in Table 1 (young CI users) and Table 2 (elderly CI users). Apart from two CI users, all of the implanted individuals were *postlingually deafened*. This was defined by the time period of normal hearing ability in the (later implanted) ear or a sufficient acoustic hearing albeit with enhanced hearing threshold and (frequently) supported by a hearing aid before deafness and cochlear implantation. If the subjects were able to hear acoustically and to acquire spoken language before the age of 3.5 years, they were assumed to be *postlingually deafened* individuals (the time point of *deafness* was *after* language acquisition). The remaining two CI users were *prelingually deafened*, as defined by the onset of *deafness before* the age of 3.5 years. Importantly, however, these two individuals were implanted *early* in life (i.e., at the age of 1 and 3 years; see Table 1), which allows a normal development of the central auditory system (Sharma et al., 2002). None of the implanted individuals used sign language as a main communication channel. The CI users were either implanted unilaterally (young: *N* = 6, elderly: *N* = 5) or bilaterally (young: *N* = 7, elderly: *N* = 8). All participants had been using the tested CI continuously for at least 12 months before study participation (mean use and SD: 10.6 ± 8.8 years, range: 1–27 years). In the case of bilateral implantation, the subjectively better CI ear was tested, which was the first implanted ear side in most of the CI users (*N* = 24). As far as possible, all preprocessing strategies of the CI sound processor were switched off to avoid any confounding influence of CI-related noise suppression on cortical speech processing. See Table 1 (young CI users) and Table 2 (elderly CI users) for further details about the participants with CI.

**TABLE 1:** Demographic variables of the young participants with CI.

ID	Age	Sex	Stimulated CI ear	Contra-lateral ear	Processor information (CI side)	Etiology	Age at implantation of stimulated ear (years)	CI experience (years)	WHO grade in contralateral ear (dB)	GöSa quiet (%)	GöSa noise (dB)
CI_J_01	25	F	Left	HA	Opus2	Unknown	7	18	4	87.00	5.70
CI_J_02	37	F	Left	HA	Kanso Soundprocessor, CP950	Unknown	35	2	4	93.30	4.40
CI_J_03	25	F	Right	CI	SONNET	Unknown	5	21	4	90.40	2.50
CI_J_04	19	F	Right	CI	SONNET	Genetically	14	5	4	79.80	5.80
CI_J_05	29	F	Right	CI	CP910	Genetically	5	24	4	95.00	3.90
CI_J_06	24	M	Left	CI	Opus2	Genetically	4	20	4	90.60	2.50
CI_J_07	24	F	Left	CI	CP910	Unknown	16	8	4	99.40	1.90
CI_J_08	32	M	Right	Unprovided	CP910	Explosion trauma	5	27	4	65.50	7.80
CI_J_09	26	M	Right	HA	SONNET	Unknown	4	22	4	97.30	2.00
CI_J_10	23	F	Right	HA	CP1000	Genetically and meningitis*	3	20	4	89.10	3.00
CI_J_11	22	F	Right	CI	CP1000	Unknown	21	1	4	98.40	0.80
CI_J_12	25	M	Right	Unprovided	CP910	Premature birth	5	20	4	68.00	10.30
CI_J_13	20	F	Right	CI	Naida CI Q90	Congenital*	1	19	4	95.20	2.40

*Note that these patients were prelingually deaf, but implanted before an age of 3.5 years.

**TABLE 2:** Demographic variables of the elderly participants with CI.

ID	Age	Sex	Stimulated CI ear	Contra-lateral ear	Processor information (CI side)	Etiology	Age at implantation of stimulated ear (years)	CI experience (years)	WHO grade in contralateral ear (dB)	GöSa quiet (%)	GöSa noise (dB)
CI_A_01	73	F	Left	CI	NaidaCIQ90	Genetically	72	1	4	87.10	6.20
CI_A_02	63	F	Left	CI	SONNET EAS	Unknown	61	2	4	86.10	8.10
CI_A_03	76	F	Left	CI	SONNET	Genetically	71	4	4	79.80	6.10
CI_A_04	63	M	Left	CI	CP1000	Unknown	62	1	4	77.20	7.00
CI_A_05	78	F	Right	CI	CP1000	Unknown	65	13	4	86.30	2.20
CI_A_06	64	M	Left	HA	SONNET	Unknown	60	4	4	95.70	−0.50
CI_A_07	60	M	Right	Un-provided	CP910	Genetically	44	16	4	61.20	5.30
CI_A_08	71	F	Right	CI	SONNET	Sudden deafness	69	2	4	91.60	3.00
CI_A_09	77	F	Right	HA	CP810	Unknown	71	7	4	92.70	3.50
CI_A_10	69	F	Left	CI	SONNET EAS	Unknown	66	3	4	85.00	2.80
CI_A_11	77	M	Left	CI	SONNET	Genetically	68	10	4	95.40	4.60
CI_A_12	74	F	Left	HA	SONNET EAS	Unknown	71	3	3	76.80	4.30
CI_A_13	79	F	Right	HA	SONNET EAS	Morbus Meniére	77	3	2	49.50	8.10

CI users and NH listeners were matched by gender, age, handedness, and years of education (mean education and SD: NH young: 17.3 ± 1.8 years, range: 15–20 years; NH old: 15.6 ± 3.2 years, range: 10–20 years; CI young: 15.2 ± 2.9 years, range: 9–20 years; CI old: 13.5 ± 3.5 years, range: 4–18 years). Additionally, within each age group, the stimulation side was matched between CI users and NH listeners (young: 4 left side, elderly: 8 left side). All of the participants were right-handed (Edinburgh inventory; range: 80–100%; Oldfield, 1971), they had normal or *corrected-to normal* vision, and they were predominantly native German speakers or had a comprehensive knowledge of German. None of the participants exhibited a neurological disease or used psychotropic drugs. All of the elderly participants showed a normal, age-appropriate cognition, as indicated by the scores in the Mini-Mental State Test (mean points and SD of elderly CI users *and* NH listeners: 28.46 ± 1.68 points, range: 24–30; total score: 30 points, score of 20 or less: hint for dementia; Folstein et al., 1975).

In the NH controls, the normal hearing threshold was confirmed by pure tone audiometry, which revealed ≤ 25 dB mean hearing loss averaged over the tested frequencies 500, 1,000, 2,000, and 4,000 Hz (4 pure tone average, 4PTA).

All participants gave written informed consent before study participation and were reimbursed. The study was approved by the local Ethics Committee of the medical faculty of the University of Cologne (application number: 18-197) and was conducted in accordance with the World Medical Association (2013).

### Electroencephalography paradigm and stimuli

**FIGURE 1:**
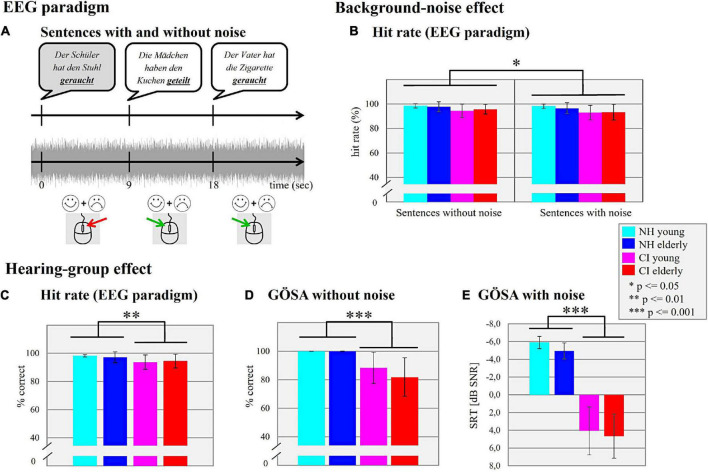
Electroencephalography (EEG) paradigm. **(A)** Sentences with and without noise. Schematic illustration of the sentence paradigm used in the actual study. By pressing a relevant mouse button, the participants were asked to distinguish between semantically correct (white) and semantically incorrect (gray; i.e., violated) sentences. Background-noise effect. **(B)** Hit rate (EEG paradigm). The mean hit performance (%) of the four different subgroups (NH young, NH elderly, CI young, CI elderly) in the situation without and with background noise is shown. Hearing-group effect. **(C)** Hit rate (EEG paradigm). The mean hit perfo rmance (%) for the four different subgroups is shown independently of the background noise. **(D)** GÖSA without noise. Mean percentage of the correctly identified words (% correct) for each subgroup is shown. **(E)** GÖSA with noise. Mean SRT (dB SNR) for a 50% speech intelligibility for each subgroup is shown.

The participants performed an auditory sentence categorization task (Hahne et al., 2012) including semantically correct and incorrect (i.e., violated) sentences presented either with or without background noise. The sentences consisted of six words (determinative, subject, the auxiliary “hat/haben” = “has/have,” determinative, object, past participle), and they were uttered in a moderate tempo by a female speaker (213 ± 93 ms between words). The final word of each German sentence was either semantically correct (e.g., “Der Vater hat die Zigarette geraucht”/“The father has smoked the cigarette”) or incorrect (e.g., “Der Schüler hat den Stuhl geraucht”/“The student smoked the chair”) with regard to the previous sentence context (Figure 1A). Notice, that only in German the critical word is also the last word in each sentence (as used in the current study); in the English translation, the critical word would be in the middle of each sentence. Given that the sentences were naturally spoken, the sentence durations (mean and SD: 4,935 ± 356 ms, range: 4,014–5,591 ms) and the latencies of the sentence-final words were variable (mean and SD: 4,011 ± 341 ms, range: 3,243–4,824 ms). The mean trial duration was 9,795 ± 356 ms (range: 8,874–10,451 ms), and the interstimulus-interval was set at 4,860 ms.

In total, the stimulus material consisted of 80 sentences (used for EEG recording) and seven training sentences (used for familiarization with the task). The Presentation software (Neurobehavioral Systems, version 21.1) was used to present the stimuli. Each trial started with a white fixation cross presented visually on a black background on the screen, which was followed by the sentence onset 1,000 ms later. The fixation cross persisted on the screen over the entire duration of auditory stimulation. The participants were instructed to judge the sentences in terms of semantic congruity. Importantly, all of the sentence-final words were presented once in a semantically correct sentence and once in a semantically incorrect sentence. This guaranteed that the resulting N400 event-related potential (ERP) effects (assessed by comparing the ERPs between the correct and incorrect sentences) were not confounded by physical stimulus properties but were specifically related to cognitive-linguistic integration processes. They were asked to respond by pressing the relevant button of a computer mouse as soon as they perceived a response signal on the screen (white smileys on black background) that was presented directly after the sentence offset.

All participants were tested in an electromagnetically shielded and sound attenuated room while sitting in a recliner in front of a computer screen. The sentences were presented either with or without stationary background noise (ICRA noise; Dreschler et al., 2001). Importantly, the same stationary noise was also used in the speech audiometry (Göttingen Sentence Test with noise; see section “Additional behavioral tests” for more details), which allowed a direct comparison between the performance in the EEG task and the speech intelligibility assessed by a clinical speech test.

Before each condition, the participants were accustomed to the task by a short training session. The experimental session was structured in four recording blocks (two with and two without background noise), containing 40 different sentences each and interrupted with short breaks in between. The block order was randomized across the participants and the order of the sentences was pseudo-randomized. In total, the recording time was about 26 min.

In the two NH groups (young and elderly), the auditory stimuli were presented monaurally through an insert earphone (3M E-A-RTONE 3A ABR), on the same side as the matched CI user. The contralateral ear was closed by means of an earplug. For the two CI user groups (young and elderly), the auditory stimuli were presented via a loudspeaker (LAB 501, Westra Electronic GmbH) placed in front of the participants (S0N0). To avoid possible confounding effects by the second ear, the contralateral device (hearing aid or second CI) was removed during the recording, and the ear was additionally blocked with an earplug.

The sentences were presented with a sound intensity of 65 dB SPL. All participants rated the perceived loudness of the sentences by means of a seven-point loudness rating scale, which is usually used in clinical context, and which allowed to adjust the sound intensity to a moderate level if necessary (Allen et al., 1990; Zeng, 1994; Sandmann et al., 2015). Thus, the stimulus loudness was comparable across all participants and corresponded to a moderate sound intensity of 60–70 dB SPL. The background noise was set at a 10 dB signal-to-noise ratio (SNR).

In order to analyze the behavioral data, we calculated the overall hit rate that is the percentage of the correctly categorized sentences. This hit rate was determined separately for each participant and each condition (with/without background noise).

### Additional behavioral tests

The participants were checked for their hearing status, including the *pure-tone audiometry* for NH listeners and the “*Aufblähkurve*” (aided threshold) for CI users (4PTA over 500, 1,000, 2,000, and 4,000 Hz) and the *Göttingen Sentence Test* (GÖSA; Kollmeier and Wesselkamp, 1997) performed once with ICRA background noise (Dreschler et al., 2001) and once without background noise (65 dB speech intensity). The GÖSA with background noise is an adaptive measurement to calculate a speech recognition threshold (SRT; dB value at a 50% speech intelligibility). In our study, we used a constant background noise at 65 dB SPL and variable sound levels for the speech stimuli.

Following previous studies, the participants performed a behavioral lip-reading task (Stropahl et al., 2015; Stropahl and Debener, 2017). It was shown that CI users may develop strategies to compensate the auditory deprivation due the hearing loss (before implantation) or to compensate the limited auditory input by the electrical hearing with a CI (after implantation; Rouger et al., 2007; Lazard et al., 2012a). Indeed, previous results point to an enhanced lip-reading ability in CI users compared to NH listeners (e.g., Layer et al., 2022). In the current study, we used a lip-reading task including purely visual words (*N* = 42) from the Freiburg monosyllabic speech test (Hahlbrock, 1970). The words were articulated by three different speakers (two females, one male) and they were taken from the Oldenburg Audio Visual Speech Stimuli (OLAVS) pool (Stropahl et al., 2017). The participants were instructed to repeat the words they perceived on the basis of the lip movements. The overall hit rate was calculated as the percentage of the correctly recognized words.

In order to explore whether CI users show comparable age effects on cognition as NH listeners (e.g., Salthouse, 1996; Park et al., 2002), we tested the participants with four established neuropsychological tests. To avoid possible confounds caused by hearing impairment, all of these tests were performed in non-auditory conditions, and they contained comprehensive written instructions. The first cognitive test was the German version of the *Size Comparison-Span test* (SICSPAN; Sörqvist et al., 2010) which was used to investigate the capacity of verbal working memory. This test is a visual computer-based task, where the participants have to respond to different questions and simultaneously have to memorize words that are presented between the questions. The percentage of the correctly remembered words was calculated and used for the statistical analyses. The second cognitive test was a German multiple-choice vocabulary test (“*Mehrfachwahl-Wortschatz-Intelligenz-Test*,” MWT-B; Lehrl et al., 1995) where the participants were asked to recognize the real word from a series of unreal words in each row (37 rows). The number of correctly identified real words (max. 37) was used for the statistical analyses. This vocabulary test captures the crystalline verbal intelligence (Lehrl, 2005). The third cognitive test was the *Trail Making Test* (TMT; Reynolds, 2002) to examine the cognitive flexibility (Arbuthnott and Frank, 2000; Hester et al., 2005). The participants should join numbers (TMT-A) or numbers and letters (TMT-B) by ascending order as fast as possible. The difference between the TMT-B and TMT-A was calculated and used for the statistical analyses. The fourth cognitive test was the *verbal learning and verbal retrieval* test from the German Version of “Consortium to Establish a Registry for Alzheimer’s Disease” (CERAD)-Plus Neuropsychological Test Battery from Memory Clinic Basel (CERAD-Plus Test Battery Memory Clinic Basel, 2022)^1^. In the verbal learning test, the participants were instructed to read a word list (consisting of 10 words) aloud and to immediately repeat all of these words they could remember. This procedure was repeated twice (=verbal learning). After a delay of around 5 min, the subjects were asked to report as many words from the list as they remembered without reading the list again (=verbal retrieval). The percentage of the correctly remembered words was calculated separately for verbal learning and verbal retrieval.

### Recording and analysis of electrophysiological data

#### Electroencephalography data recording

For the EEG recording, 31 Ag/AgCl ActiCap slim electrodes were used (BrainProducts, 2022 Gilching, Germany) which were placed according to the international 10/20 system by means of a customized electrode cap (Easycap, Herrsching, Germany). One additional electrode was placed below the left eye for the recording of the electrooculogram (EOG). The ground electrode was chosen slightly anterior to the Fz electrode. The EEG was continuously recorded and amplified by using a BrainAmp DC amplifier (BrainProducts, 2022)^2^. All channels were recorded against the reference electrode localized on the tip of the nose. The impedance of each electrode was kept below 5 kΩ during the recordings. The EEG data was digitized with a sampling rate of 1,000 Hz and was online analogically filtered between 0.02 and 250 Hz.

#### Electroencephalography preprocessing

The EEG data was analyzed by using EEGLAB (version 2019_1; Delorme and Makeig, 2004) running in the MATLAB environment (R2018b, Mathworks). Only the trials with correct behavioral responses (semantically correct/incorrect sentence) were included into the further ERP analyses. The raw EEG data was downsampled to 500 Hz and filtered offline by using a Finite Impulse Response (FIR) filter. A high pass cut-off frequency of 0.1 Hz and a low-pass cut-off frequency of 40 Hz was used, with a transition bandwidth of 0.2 and 2 Hz, respectively. For both filters, the Kaiser-window approach was applied (beta = 5.653, max. stopband attenuation = −60 dB, max. passband deviation = 0.001, transition width normalized freq = 3.6/m), which allowed to maximize the energy concentration in the main lobe and to minimize the information loss at the edges of the window (Widmann et al., 2015). In CI users, the EEG channels located at the speech processor and the CI transmitter coil were removed (mean and SD: 1.5 ± 1 electrodes; range: 0–4 electrodes). Afterward, the data of all participants was additionally filtered (high-pass 1 Hz) and segmented into 2-s epochs, and bad epochs containing unique, non-stereotype artifacts were eliminated by using a joint probability approach (function *jointprob.m*; threshold criterion: four standard deviations). In a next step, an independent component analysis (ICA) was applied on the epoched data (Bell and Sejnowski, 1995), and the resulting ICA weights were applied to the originally filtered (0.1–40 Hz) and epoched data (−200 to 7,998 ms relative to the sentence onset). Independent components (ICs) representing artifacts caused by eye movements, electrical heartbeat activity, and other non-cerebral activity were identified and removed from the data (Jung et al., 2000). In addition, following the procedures in previous studies (Debener et al., 2008; Sandmann et al., 2009, 2010; Schierholz et al., 2017) we identified and removed the ICs accounting for the electrical CI artifact by means of the centroid on the side of the CI device, and by the time course of the component activity, showing maximal activation from 30 to 110 ms after sentence onset. The overall number of rejected ICs was 12.46 ± 2.40 (mean ± 1 standard deviation) for the young CI users, 9.85 ± 2.73 for the elderly CI users, 7.15 ± 2.38 for the young NH listeners, and 9.15 ± 1.77 for the elderly NH. Afterward, the missing channels in the CI users (located at the speech processor and the transmitter coil) were interpolated by using a spherical spline interpolation (Perrin et al., 1989). Single-subject ERPs to the onset of the sentence (−200 to 7,998 ms) and the sentence-final word (−200 to 2,798 ms) were computed, separately for the conditions with and without background noise. The resulting ERPs were baseline corrected (−200 to 0 ms relative to the onset of the sentence or sentence-final word). In addition, a difference wave was computed for the ERPs to the sentence-final word in order to assess the N400 effect, separately for the conditions with and without background noise (N400 effect = ERP amplitude of semantically incorrect sentences – ERP amplitude of semantically correct sentences). In this difference wave, we also computed the N400 latency (see also next section). After preprocessing, the percentage of residual trials (with correct behavioral responses) was for the young CI users 85 ± 6%, for the elderly CI users 85 ± 5%, for the young NH listeners 88 ± 4% and for the elderly NH listeners 87 ± 3%.

#### Event-related potential data analysis

We performed a peak detection analysis on single-subject ERPs. This was done for the N1 and P2 ERPs in response to the sentence onset (reflecting sensory-driven processes), and for the N400 ERP (difference waveform) in response to the sentence-final word (reflecting cognitive-linguistic integration processes). We used a frontocentral region-of-interest (ROI; F3, Fz, F4, FC1, FCz, FC2, C3, Cz, C4) for the N1 and P2 ERPs, and a frontocentroparietal ROI (F3, Fz, F4, FC1, FCz, FC2, C3, Cz, C4, CP1, Pz, CP2) for the N400 ERP. Using several channels in the ROIs is advantageous because it results in an improved SNR for the ERPs, and it takes the variability across individuals with regard to the specific channel showing the strongest ERP responses into account. In general, the ROIs and latency windows of ERPs (N1: 50–190 ms; P2: 130–430 ms; N400: 400–1,200 ms) were defined based on the grand average computed across all conditions and participants.

The ERP amplitudes were quantified by means of the signed area, that is, the positive (P2) and the negative (N1, N400) area under the (ERP) curve at the respective latency windows. Regarding the ERP latencies, we computed the 50% fractional-area latency measure separately for each ERP (N1, P2, N400). This was done by computing the (total) signed area under the respective ERP waveform and by finding the latency at which 50 percent of this ERP-specific area are reached (Hansen and Hillyard, 1980; Kiesel et al., 2008; Luck, 2014). The use of the area amplitude and the fractional-area latency measures are advantageous compared to the more conventional ERP peak measures because it allows more accurate estimates of ERP parameters, with greater statistical power and no inflation of the Type I error rate (Kiesel et al., 2008). Moreover, the linear fractional-area measure is not influenced by single-trial latency jitter and is relatively insensitive to high-frequency noise (Petermann et al., 2009; Meyer et al., 2011; Luck, 2014).

### Statistical analyses

In general, the statistical analyses were performed in SPSS (IBM SPSS Statistics, Version 27.0.0.0). To analyze the performance in the sentence categorization task (EEG paradigm), we computed a 2 × 2 × 2 mixed ANOVA, with “condition” (with and without background noise) as the within-subjects factor, and the factors “hearing” (NH listeners and CI users) and “age” (young and elderly) as the between-subjects factors. Similarly, we computed 2 × 2 × 2 mixed ANOVAs for the ERP amplitude and latency measures, separately for the N1, P2 and N400 ERPs. In the case of violation of the sphericity assumption, a Greenhouse-Geisser correction was applied. In general, significant interaction effects (*p* ≤ 0.05) were followed-up with *post-hoc t*-tests, and a Holm-Bonferroni correction (Holm, 1979) was applied to correct for multiple comparisons. The partial eta square (η*_*p*_*^2^), and Cohen’s d were reported as a measure of effect size.

Regarding the additional behavioral measures, as assessed by the lip-reading test and the cognitive tests (working memory, verbal intelligence, cognitive flexibility, verbal learning and retrieval), we computed a multifactorial ANOVA (without measurement repetition), with the fixed factors “age” (young/elderly) and “hearing” (NH/CI) separately for each test. Similarly, a multifactorial ANOVA was also used for the measures obtained by the clinical speech tests, in particular the Göttingen Sentence Test (GÖSA) performed once with and once without background noise. In the case of statistically significant interaction effects (*p* ≤ 0.05), *post-hoc t*-tests were performed and corrected for multiple comparisons by using the Holm-Bonferroni correction (Holm, 1979).

We expected a correlation of the hit rate in the EEG paradigm in the present study with the speech intelligibility assessed by the clinical speech tests. Therefore we computed non-parametric Spearman’s rank correlations (Spearman, 1904) between the hit rates of the EEG paradigm (sentence categorization task) and the results of the GÖSA, separately for the conditions with and without background noise. Additionally, we expected a relationship between WMC and speech-in-noise processing in young and elderly CI users. Specifically, we computed non-parametric Spearman’s rank correlations between the WMC and the N400 component (amplitude and latency) separately for both listening conditions (with and without background noise) in the CI users across both age ranges. In general, for all analyses we reported only the original, uncorrected *p*-values.

## Results

### Behavioral results of the sentence categorization task (electroencephalography paradigm)

In general, all groups of participants achieved high performance levels in the sentence categorization task, both in the conditions with and without background noise (Figure 1B). The 2 × 2 × 2 mixed ANOVA with “condition” (with/without background noise) as within-subject factor and the factors “age” (young/elderly) and “hearing” (CI/NH) as the between-subject factors showed no main effect of “age” (*F*_1_,_48_ = 0.017, *p* = 0.898, $\eta_{p}^{2}$ = 0.001), but a significant main effect of “hearing” (*F*_1_,_48_ = 10.433, *p* = 0.002, $\eta_{p}^{2}$ = 0.179), which was due to a higher performance level in the NH listeners when compared to the CI users (mean ± SD of NH: 97.72 ± 2.79%; mean ± SD of CI: 94.11 ± 4.88%; Figure 1C). Furthermore, the 2 × 2 × 2 mixed ANOVA revealed a significant main effect of “condition” (*F*_1_,_48_ = 5.943, *p* = 0.019, $\eta_{p}^{2}$ = 0.110), which was caused by lower speech performance in the condition with (mean hit rate ± SD: 95.22 ± 5.30%) compared to without background noise (mean hit rate ± SD: 96.61 ± 4.23%).

### Results of speech audiometry, lip-reading ability, and cognitive tests

The results of the *speech audiometry* performed with the GÖSA are shown in Figure 1D (without background noise) and in Figure 1E (with background noise). The multifactorial ANOVA computed with the factors “age” (young/elderly) and “hearing” (CI/NH) revealed for the GÖSA *without* background noise no significant main effect for the factor “age” (*F*_1_,_48_ = 1.843, *p* = 0.181, η*_*p*_*^2^ = 0.037). However, there was a significant main effect for the factor “hearing” (*F*_1_,_48_ = 37.930, *p* = 0.001, η*_*p*_*^2^ = 0.441), which was caused by a higher speech recognition ability in the NH listeners (mean ± SD: 99.98 ± 0.10%) when compared to the CI users (mean ± SD: 85.13 ± 12.50%). No significant interaction effect between “age” and “hearing” was observed (*F*_1_,_48_ = 1.800, *p* = 0.186, η*_*p*_*^2^ = 0.036). Regarding the GÖSA performed *with* background noise, there was neither a significant main effect of “age” (*F*_1_,_48_ = 2.105, *p* = 0.153, η*_*p*_*^2^ = 0.042) nor a significant “age” × “hearing” interaction (*F*_1_,_48_ = 0.115, *p* = 0.736, η*_*p*_*^2^ = 0.002). As expected, however, we found a significant main effect of “hearing” (*F*_1_,_48_ = 338.081, *p* = 0.001, η*_*p*_*^2^ = 0.876), which was caused by lower SRTs in NH listeners (mean ± SD: −5.42 ± 0.93 dB) than in CI users (mean ± SD: 4.37 ± 2.56 dB).

**FIGURE 2:**
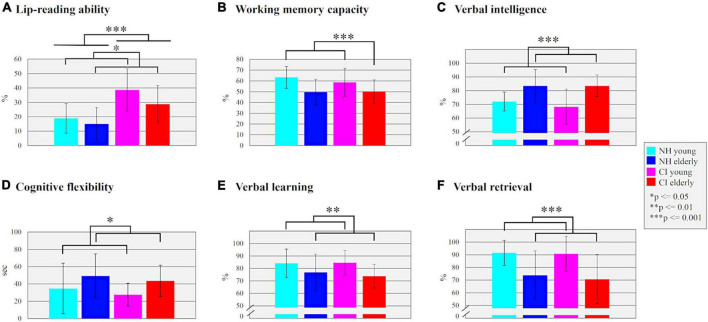
**(A)** Lip-reading ability. The mean performance (%) of correctly read words for each subgroup is shown. **(B)** Working memory capacity. The mean performance (%) of correctly remembered words in the *Size Comparison-Span test* is shown as a measure for working memory capacity. **(C)** Verbal intelligence. The mean performance (%) in the multiple-choice vocabulary test (“Mehrfachwahl-Wortschatz-Intelligenz-Test”) is compared between the subgroups. **(D)** Cognitive flexibility. The difference between the *Trail Making Tests* **(A,B)** in seconds is compared between all subgroups. **(E)** Verbal learning. The mean performance (%) of correctly remembered words for each subgroup is shown. **(F)** Verbal retrieval. The mean performance (%) of correctly remembered words after a delay of around 5 min was compared between all subgroups.

The results of the *lip-reading ability* are illustrated in Figure 2A. The multifactorial ANOVA computed with the factors “age” (young/elderly) and “hearing” (CI/NH) revealed a significant main effect “hearing” (*F*_1_,_48_ = 23.473, *p* = 0.001, η*_*p*_*^2^ = 0.328), which was due to enhanced lip-reading ability in CI users (mean ± SD: 33.70 ± 14.52%) when compared to NH listeners (mean ± SD: 16.94 ± 10.82%). Moreover, there was a (marginally) significant main effect of “age” (*F*_1_,_48_ = 3.942, *p* = 0.053, η*_*p*_*^2^ = 0.076), which originated from enhanced lip-reading ability in young individuals (mean ± SD: 28.76 ± 16.03%) when compared to the elderly ones (mean ± SD: 21.89 ± 13.87%). The “age” × “hearing” interaction was not significant (*F*_1_,_48_ = 0.763, *p* = 0.387, η*_*p*_*^2^ = 0.016).

The results of the different *cognitive tests* are shown in Figures 2B–F. In the *SICSPAN* (Figure 2B), which examines the *WMC*, the measurement had to be aborted in one of the elderly CI users (ID: CI_A_13) due to personal excessive demands. The multifactorial ANOVA computed with the factors “age” (young/elderly) and “hearing” (CI/NH) revealed a main effect of “age” (*F*_1_,_47_ = 11.779, *p* = 0.001, η*_*p*_*^2^ = 0.200), with younger participants (mean ± SD: 60.99 ± 11.62%) showing better WMC than elderly individuals (mean ± SD: 49.89 ± 11.20%). However, there was no main effect of “hearing” (*F*_1_,_47_ = 0.397, *p* = 0.532, η*_*p*_*^2^ = 0.008) or any interaction effect (*F*_1_,_47_ = 0.590, *p* = 0.446, η*_*p*_*^2^ = 0.012).

The multifactorial ANOVA with the *verbal intelligence test* (Figure 2C) as dependent variable revealed a significant main effect of “age” (*F*_1_,_48_ = 22.196, *p* = 0.001, η*_*p*_*^2^ = 0.316), with elderly individuals showing an increased verbal intelligence (mean ± SD: 83.58 ± 9.91%) compared to young individuals (mean ± SD: 70.27 ± 10.23%). There was neither an effect of “hearing” (*F*_1_,_48_ = 0.439, *p* = 0.511, η*_*p*_*^2^ = 0.009) nor a significant “age” × “hearing” interaction (*F*_1_,_48_ = 0.439, *p* = 0.511, η*_*p*_*^2^ = 0.009).

Regarding the *cognitive flexibility* (Figure 2D), we found in the multifactorial ANOVA a significant main effect of “age” (*F*_1_,_48_ = 6.213, *p* = 0.016, η*_*p*_*^2^ = 0.115), which was caused by a higher cognitive flexibility in the younger (mean ± SD = 30.50 ± 22.38 s) compared to the elderly individuals (mean ± SD = 46.01 ± 21.83 s). There was neither a significant main effect of “hearing” (*F*_1_,_48_ = 0.553, *p* = 0.461, η*_*p*_*^2^ = 0.011) nor a significant “age” × “hearing” interaction (*F*_1_,_48_ = 0.001, *p* = 0.981, η*_*p*_*^2^ = 0.001).

Finally, the multifactorial ANOVAs computed on the measures from the *verbal learning and verbal retrieval test* (Figures 2E,F) revealed significant main effects of “age” in both subtests (learning: *F*_1_,_48_ = 7.560, *p* = 0.008, η*_*p*_*^2^ = 0.136; retrieval: *F*_1_,_48_ = 17.785, *p* = 0.001, η*_*p*_*^2^ = 0.270), with enhanced verbal learning and retrieval capacities in the young individuals (learning: mean ± SD: 84.10 ± 10.26%; retrieval: mean ± SD: 91.15 ± 11.77%) when compared to the elderly individuals (learning: mean ± SD: 75.38 ± 12.19%; retrieval: mean ± SD: 72.31 ± 19.04%). There were no significant effects of “hearing” (learning: *F*_1_,_48_ = 0.105, *p* = 0.748, η*_*p*_*^2^ = 0.002; retrieval: *F*_1_,_48_ = 0.185, *p* = 0.669, η*_*p*_*^2^ = 0.004) and no significant “age” × “hearing” interactions (learning: *F*_1_,_48_ = 0.418, *p* = 0.521, η*_*p*_*^2^ = 0.009; retrieval: *F*_1_,_48_ = 0.067, *p* = 0.797, η*_*p*_*^2^ = 0.001).

Taken together, the results revealed reduced behavioral speech performance for the CI users (Figures 1C–E) but enhanced lip-reading ability (Figure 2A) when compared to the NH listeners. Further, the cognitive tests revealed consistent age effects across both the CI users and the NH listeners. When compared to younger individuals, elderly individuals showed higher verbal intelligence (Figure 2C), but decrements in working memory (Figure 2B), cognitive flexibility (Figure 2D), verbal learning (Figure 2E) and verbal retrieval (Figure 2F).

### Event-related potential results: Sensory-driven processes (N1 and P2)

**FIGURE 3:**
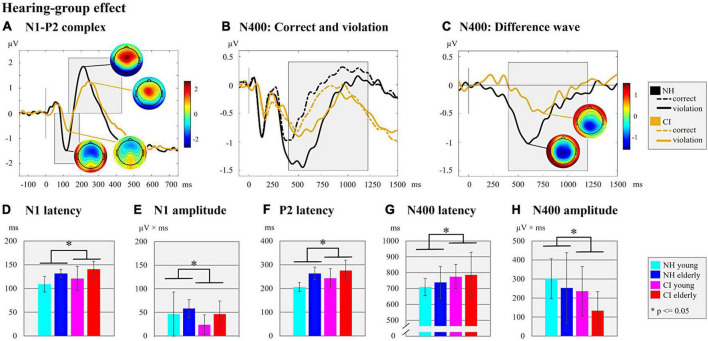
Hearing-group effect. **(A)** N1-P2 complex. ERPs are shown for the two hearing groups (NH/CI) independent of the background noise (with/without) and the age (young/elderly). The ERP topographies at the N1 (CI user: 128 ms; NH listeners: 116 ms) and P2 peaks (CI user: 238 ms; NH listeners: 212 ms) are given separately for each hearing group. Gray-shaded boxes indicate the N1 and P2 time windows for peak and latency detection. **(B)** N400: Correct and violation. ERPs are shown for the two hearing groups (NH/CI) independent of the background noise and age but separately for the different semantic context of the sentences (semantically correct vs. semantically incorrect/violation). Gray-shaded box indicate the N400 time window for the peak and latency detection of the supplementary analyses. **(C)** N400: Difference wave. The difference waves of the N400 ERP are shown for each hearing group (NH/CI) independent on the background noise and age. The ERP topographies at the N400 peaks are shown for each hearing group (CI user: 750 ms; NH listeners: 600 ms). The Gray-shaded box indicates the N400 time window for the peak and latency detection. **(D)** N1 latency. Bar plots with mean N1 latency (ms) for each subgroup. **(E)** N1 amplitude. Bar plots with mean N1 amplitude (μV × ms) for each subgroup. **(F)** P2 latency. Bar plots with mean P2 latency (ms) for each subgroup. **(G)** N400 latency. Bar plots with mean N400 latency of the difference waves (ms) for each subgroup **(H)** N400 amplitude. Bar plots with mean N400 amplitude area of the difference waves (μV × ms) for each subgroup.

**FIGURE 4:**
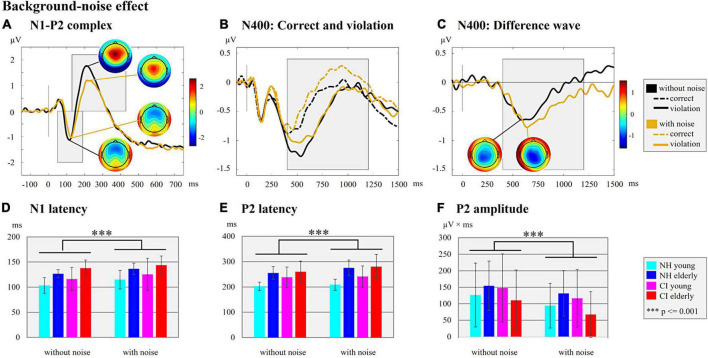
Background-noise effect. **(A)** N1-P2 complex. ERPs are shown for the hearing condition with and without background noise independent of the hearing group (NH/CI) and the age (young/elderly). The ERP topographies at the N1 (without background noise: 116 ms; with background noise: 124 ms) and P2 peaks (without background noise: 210 ms; with background noise: 214 ms) are given separately for each hearing condition (with/without background noise). Gray-shaded boxes indicate the N1 and P2 time windows for peak and latency detection. **(B)** N400: Correct and violation. ERPs are shown for the two hearing conditions with and without background noise independent of the factors hearing and age but separately for the different semantic context of the sentences (semantically correct vs. semantically incorrect/violation). The gray-shaded box indicates the N400 time window for the peak and latency detection of the supplementary analyses. **(C)** N400: Difference wave. The difference waves of the N400 ERP are shown for each hearing condition (with/without background noise) independent of the hearing groups and age groups. The ERP topographies at the N400 peaks are shown for both hearing conditions (without background noise: 588 ms; with background noise 638 ms). The Gray-shaded box indicates the N400 time window for the peak and latency detection. **(D)** N1 latency. Bar plots with mean N1 latency (ms) for each subgroup separately for the hearing condition with and without background noise. **(E)** P2 latency. Bar plots with mean P2 latency (ms) for the comparison between each subgroup separately for the hearing condition with and without background noise are shown. **(F)** P2 amplitude. Bar plots with mean P2 amplitude area (μV × ms) for each subgroup separately for each hearing condition (with/without background noise) are shown.

**FIGURE 5:**
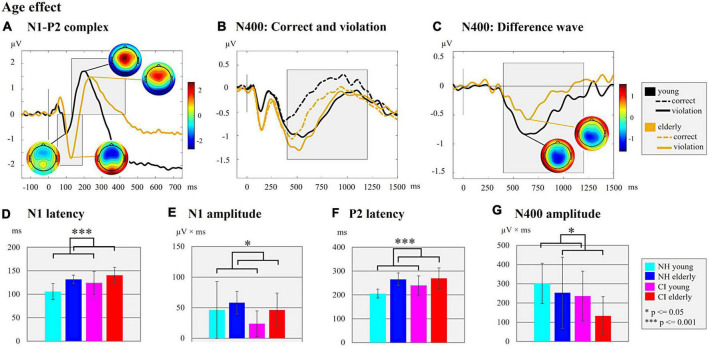
Age effect. **(A)** N1-P2 complex. ERPs are shown for the two age groups (young/elderly) independent of background noise and of the hearing groups (NH/CI). The ERP topographies at the N1 (young individuals: 100 ms; elderly individuals: 126 ms) and P2 peaks (young individuals: 200 ms; elderly individuals: 236 ms) are given separately for each age group. Gray-shaded boxes indicate the N1 and P2 time windows for peak and latency detection. **(B)** N400: Correct and violation. ERPs are shown for the two age groups (young/elderly) independent on background noise and the hearing groups but separately for the different semantic context of the sentences (semantically correct vs. semantically incorrect/violation). The gray-shaded box indicates the N400 time window for the peak and latency detection of the supplementary analyses. **(C)** N400: Difference wave. The difference waves of the N400 ERP are shown for each age group (young/elderly) independent of the background noise and the hearing groups. For both age groups the ERP topographies at the N400 peaks are shown (young individuals: 622 ms; elderly individuals: 644 ms). The Gray-shaded box indicates the N400 time window for the peak and latency detection. **(D)** N1 latency. Bar plots with mean N1 latency (ms) for each subgroup independent of the background noise are shown. **(E)** N1 amplitude. Bar plots with mean N1 amplitude area (μV × ms) for each subgroup are shown. **(F)** P2 latency. Bar plots with mean P2 latency (ms) for the comparison between each subgroup independent of background noise are shown. **(G)** N400 amplitude. Bar plots with mean N400 amplitude area of the difference wave (μV × ms) for each subgroup are compared.

The grand averages of the N1 and P2 ERPs elicited to the sentence onset are given in the Figures 3A, 4A, 5A. These figures show the effect of the hearing group (CI vs. NH; Figure 3), the effect of background noise (with vs. without; Figure 4), and the effect of age (young vs. elderly; Figure 5) on N1 and P2 ERPs. The latency and amplitude of the N1 and P2 ERPs were analyzed by using a 2 × 2 × 2 mixed ANOVA, with “condition” (with/without background noise) as the within-subjects factor and “hearing” (CI/NH) and “age” (young/elderly) as the between-subjects factors, respectively. The results are presented in the following sections.

First, the results revealed *effects of the hearing group* on the N1 ERPs (Figure 3A). Specifically, we found significant main effects of the “hearing group” on the N1 latency (Figure 3D; *F*_1_,_44_ = 4.167, *p* = 0.047, η*_*p*_*^2^ = 0.087), the N1 amplitude (Figure 3E; *F*_1_,_48_ = 4.079, *p* = 0.049, η*_*p*_*^2^ = 0.078), the P2 latency (Figure 3F; *F*_1_,_47_ = 3.999, *p* = 0.051, η*_*p*_*^2^ = 0.078), but not on the P2 amplitude (*F*_1_,_48_ = 0.540, *p* = 0.466, η*_*p*_*^2^ = 0.011). These main effects were due to longer N1 and P2 latencies and smaller N1 amplitude in the CI users when compared to the NH listeners.

Further, we found *effects of background noise* on N1 and P2 ERPs (Figure 4A). In particular, we found significant main effects of the “condition” on the N1 latency (Figure 4D; *F*_1_,_44_ = 25.941, *p* = 0.001, η*_*p*_*^2^ = 0.371), the P2 latency (Figure 4E; *F*_1_,_47_ = 23.690, *p* = 0.001, η*_*p*_*^2^ = 0.335), and the P2 amplitude (Figure 4F; *F*_1_,_48_ = 18.444, *p* = 0.001, η*_*p*_*^2^ = 0.278). These main effects were caused by a prolongation of the N1 and P2 latencies and a reduction in P2 amplitude for the condition with background noise when compared to the condition without background noise. There was also a significant “condition” × “age” effect for the P2 latency (*F*_1_,_47_ = 9.628, *p* = 0.003, η*_*p*_*^2^ = 0.170). The *post-hoc* paired *t*-tests comparing the age groups (young vs. elderly) in the condition with [*t(49)* = −4.877, *p* = 0.001, *Cohen’s d* = −1.366] and the condition without background noise [*t(50)* = −3.861, *p* = 0.001, *Cohen’s d* = −1.071] revealed that a prolongation of the P2 latency specifically was pronounced in the elderly individuals when compared to the young individuals. The comparison of P2 latency between the hearing conditions within each age group (young/elderly) revealed a statistically significant prolongation of the P2 latency in the condition with background noise for the elderly individuals, but not for the young individuals [elderly individuals: *t(25)* = −5.046, *p* = 0.001, *Cohen’s d* = −0.990; young individuals: *t(24)* = −1.524, *p* = 0.141, *Cohen’s d* = −0.305].

Finally, we observed *effects of age* on N1 and P2 ERPs (Figure 5A). Specifically, there were significant main effects of “age” not only on the N1 latency (Figure 5D; *F*_1_,_44_ = 17.430, *p* = 0.001, η*_*p*_*^2^ = 0.284), but also on the N1 amplitude (Figure 5E; *F*_1_,_48_ = 4.079, *p* = 0.049, η*_*p*_*^2^ = 0.078) and the P2 latency (Figure 5F; *F*_1_,_47_ = 22.408, *p* = 0.001, η*_*p*_*^2^ = 0.323). These main effects were caused by reduced N1 and P2 latencies and smaller N1 amplitude in the young individuals when compared to the elderly individuals.

Taken together, the results revealed *effects of the hearing group* (Figure 3A) in the sensory-driven speech processes, suggesting that speech processing with a CI results in ERPs with prolonged latencies (N1 and P2) and reduced amplitudes (N1), regardless of the age and the background noise conditions. The results also showed *effects of background noise* on cortical speech processing, which were consistent in the CI users and the NH listeners (Figure 4A), and which indicated that stationary background noise results in ERPs with prolonged latencies (N1, P2) and reduced amplitudes (P2). Finally, the results revealed that cortical speech processing is also affected by the *age* in both the CI users and the NH listeners (Figure 5A), suggesting that increasing age results in ERPs with prolonged latencies (N1, P2) but enhanced ERP amplitudes (N1) in both hearing groups.

All means and standard deviations of the latencies and amplitudes (N1 and P2) for the different main effects and the interaction effects are listed in Table 3.

**TABLE 3:** Mean and SD of N1, P2 latencies and amplitudes (ERP results: Sensory-driven processes).

Effects of the hearing group	Significant main effect “hearing group”	
	CI user	N1 latency: mean ± SD: 130.80 ± 22.97 ms
		N1 amplitude: mean ± SD: 35.10 ± 26.62 μV × ms
		P2 latency: mean ± SD: 254.77 ± 44.04 ms
	NH listeners	N1 latency: mean latency and SD: 120.47 ± 17.20 ms
		N1 amplitude: mean and SD: 52.20 ± 35.31 μV × ms
		P2 latency: mean ± SD: 235.64 ± 22.69 ms

**Effects of background noise**	**Significant main effect “condition”**	

	With background noise	N1 latency: mean ± SD: 130.75 ± 22.79 ms
		P2 latency: mean ± SD: 252.20 ± 46.35 ms
		P2 amplitude: mean ± SD: 102.46 ± 75.58 μV × ms
	Without background noise	N1 latency*:* mean ± SD: 121.79 ± 20.31 ms
		P2 latency: mean ± SD: 239.76 ± 39.14 ms
		P2 amplitude: mean ± SD: 134.86 ± 91.19 μV × ms

	**Significant interaction effect “condition” × “age”**	

	With background noise	Young individuals: P2 latency: mean ± SD: 225.44 ± 37.11 ms
		Elderly individuals: P2 latency: mean ± SD: 277.92 ± 39.62 ms
	Without background noise	Young individuals: P2 latency: mean ± SD: 221.04 ± 35.85 ms
		Elderly individuals: P2 latency: mean ± SD: 257.77 ± 33.81 ms

**Effects of age group**	**Significant main effect “age”**	

	Young individuals	N1 latency: mean ± SD: 115.08 ± 22.77 ms
		N1 amplitude: mean ± SD: 35.10 ± 37.17 μV × ms
		P2 latency: mean ± SD: 222.56 ± 29.65 ms
	Elderly individuals	N1 latency: mean ± SD: 136.19 ± 14.07 ms
		N1 amplitude: mean ± SD: 52.20 ± 23.94 μV × ms
		P2 latency: mean ± SD: 267.85 ± 35.39 ms

### Event-related potential results: Cognitive-linguistic integration processes (N400)

The grand averages of the N400 ERP elicited to the sentence-final words are given in the Figures 3B,C, 4B,C, 5B,C. These figures show the effect of the hearing group (CI vs. NH; Figure 3), the effect of background noise (with vs. without; Figure 4), and the effect of age (young vs. elderly; Figure 5) on the N400 ERP. Similar to the N1 and P2 ERPs, the latency and amplitude of the N400 ERP were analyzed by using a 2 × 2 × 2 mixed ANOVA, with “condition” (with/without background noise) as the within-subjects factor and “hearing” (CI/NH) and “age” (young/elderly) as the between-subjects factors, respectively.

Regarding the *effect of hearing group* (Figures 3B,C), the results revealed a significant main effect on both the N400 latency (Figure 3G; *F*_1_,_48_ = 4.178, *p* = 0.046, η*_*p*_*^2^ = 0.080) and the N400 amplitude (Figure 3H; *F*_1_,_48_ = 6.218, *p* = 0.016, η*_*p*_*^2^ = 0.115). These main effects were caused by prolonged N400 latency and smaller N400 amplitude in the CI users (N400 latency: mean ± SD: 780.46 ± 112.40 ms; N400 amplitude: mean ± SD: 184.80 ± 124.97 μV × ms) when compared to the NH listeners (N400 latency: mean ± SD: 724.27 ± 80.58 ms; N400 amplitude: mean ± SD: 277.61 ± 149.39 μV × ms).

The data analysis revealed no significant *effect of background noise* on the N400 ERP, although the grand averages in Figures 4B,C point to (small) ERP differences between the conditions with and without background noise.

Finally, however, we observed a significant main *effect of age* (Figures 5B,C) on the N400 amplitude (Figures 5G; *F*_1_,_48_ = 4.126, *p* = 0.048, η*_*p*_*^2^ = 0.079), which was due to an enhanced N400 amplitude (mean ± SD: 269.01 ± 120.39 μV × ms) in the young individuals when compared to the elderly individuals (mean ± SD: 193.40 ± 157.95 μV × ms). There was no significant main *effect of age* on the N400 latency.

Taken together, the results revealed *effects of the hearing group* (Figures 3B,C) on the N400 ERP, suggesting that speech processing with a CI results in delayed and reduced cognitive-linguistic integration processes. Moreover, the results revealed an *effect of age* (Figures 5B,C) on the N400 ERP amplitude, which suggests that young individuals invest more neural resources for semantic integration of speech information, both in the CI users and the NH listeners.

### Results of correlation analyses

Given that the NH listeners showed ceiling effects in most of the behavioral speech measures (EEG paradigm, GÖSA without noise), the correlation analyses were restricted to the CI users. In particular, we computed non-parametric Spearman’s rank correlations between the hit rates of the EEG paradigm (sentence categorization task) and the results of the GÖSA, separately for the conditions with and without background noise, to examine the relationship between the different behavioral speech measures. The results revealed no significant correlation between the performance in the two tests for the condition *without* background noise (*r* = 0.249, *p* = 0.220; uncorrected *p*-values). However, for the tests performed *with* background noise, we observed a significant negative relationship between the hit rate in the EEG study and the speech intelligibility in the GÖSA sentence test (*r* = −0.442, *p* = 0.024; uncorrected *p*-values).

Regarding the correlation analyses between the WMC and the N400 ERP, the results failed to reach significant thresholds after Holm-Bonferroni correction. In CI users, neither the correlation between the WMC and the N400 amplitude (with background noise: *r* = 0.432, *p* = 0.031; without background noise: *r* = 0.043, *p* = 0.838; uncorrected *p*-values) nor between the WMC and the N400 latency (with background noise: *r* = 0.400, *p* = 0.048*;* without background noise: *r* = −0.035, *p* = 0.868; uncorrected *p*-values) for both listening conditions (with and without background noise) was statistically significant.

## Discussion

The current study addressed the question of how the age affects cognitive functions and the processing of sentences presented with and without background noise in CI users and in NH listeners. Our results crucially extend previous studies (Hahne et al., 2012; Henkin et al., 2014; Mosnier et al., 2015; Finke et al., 2016a; Jayakody et al., 2017; Moberly et al., 2019) by comparing not only cognitive abilities but also cortical sentence processing between two age groups (young vs. elderly) and two hearing groups (CI vs. NH), both in conditions with and without background noise.

### Hearing-group effect: Cochlear-implant users show impaired speech processing when compared to normal-hearing listeners

As expected, the CI users revealed a poorer speech recognition performance when compared to the NH listeners. Specifically, they showed reduced speech intelligibility in the clinical speech tests (GÖSA, Figures 1D,E) and lower hit rates in the sentence categorization task (EEG paradigm, Figure 1C), regardless of the presence of background noise. Our results are consistent with previous observations of limited auditory discrimination ability (Sandmann et al., 2010, 2015) and impaired speech recognition ability with a CI, even in experienced CI users (Finke et al., 2016a). In line with these behavioral findings, our ERP results showed alterations in the sensory processes of CI users when compared to NH listeners, as evidenced by prolonged (N1, P2) and reduced (N1) ERPs in the CI users (Figures 3D,E). Similarly, previous studies have reported that the N1 ERP latency of CI users is prolonged (Beynon et al., 2005; Finke et al., 2015, 2016a; Sandmann et al., 2015) and reduced in amplitude (Beynon et al., 2005; Kelly et al., 2005; Sandmann et al., 2009), pointing to impairments when speech is processed via the CI. Indeed, the CI provides only limited spectro-temporal information in a restricted dynamic range (Drennan and Rubinstein, 2008), which may explain the CI users’ difficulties to discriminate subtle changes in the acoustic properties of speech and music (Sandmann et al., 2010; Finke et al., 2016a). Importantly, the presence or absence of background noise had no effect on our observation of a hearing-group effect in both the behavioral and the EPR results across young and elderly individuals. Thus, our results extend previous research (Hahne et al., 2012; Henkin et al., 2014; Finke et al., 2016a) by demonstrating that sensory-driven processing of speech is impaired in CI users across different age groups (young and elderly) and across different listening conditions (with and without background noise).

Regarding the later cortical processing stages, our results revealed prolonged and reduced N400 ERPs in the CI users when compared with the NH listeners (Figures 3G,H). These observations are consistent with a previous study of Hahne et al. (2012), which used a sentence categorization task without background noise and which reported a prolonged N400 latency in CI users aged between 34 and 63 years in comparison to a NH control group. Our results extend these previous findings by showing both a delayed and reduced N400 response in the CI users regardless of age and presence or absence of background noise. This hearing-group effect is in line with the results on the behavioral level, which show lower hit rates in the EEG paradigm, and a reduced speech performance in the clinical speech tests (GÖSA with and without background noise) in CI users compared to NH listeners. The delayed and attenuated N400 effect in the CI users suggests that semantic processing is less effective and/or slower when speech is perceived through a CI. Speech perception via a CI is likely to require additional processing time because of the reduced availability of semantic information due to the limited CI signal. This reduced availability may affect the expectancy (regarding the critical sentence-final word; Strauß et al., 2013) and/or reduce the processing demands on the semantic integration of the sentence-final word. Indeed, a previous study with NH listeners has reported a decrease in the N400 effect for acoustically degraded compared to natural speech conditions (Aydelott et al., 2006). Our results support these previous observations by showing that our CI users, who are generally exposed to degraded CI speech signals, have a reduced N400 effect, as indicated by more similar N400 amplitudes between ERPs to semantically correct and incorrect (i.e., violated) sentences (Supplementary material and Supplementary Figures 1, 2). In contrast to our CI users, we observed a large N400 effect in our NH listeners, as indicated by significantly enhanced N400 amplitudes for the semantically incorrect compared to correct (i.e., violated) sentences (Supplementary material and Supplementary Figures 1, 2). This is consistent with the finding that NH listeners tested in undegraded speech conditions showed a significantly increased N400 effect when compared to degraded speech conditions (Aydelott et al., 2006).

We conclude that the CI users face general limitations in speech processing due to the restricted sound quality of the CI signal (in both conditions with and without background noise) which can be observed not only at earlier, sensory-driven processes (reflected by the N1-P2 complex to sentence onset) but also at later, cognitive-linguistic integration processes (reflected by the N400 to sentence-final words). These CI-related cortical alterations at various processing stages explain, in general, the CI users’ impaired speech intelligibility across different age groups and in different hearing conditions, as with and without background noise.

The degraded CI input seems to affect not only auditory but also visual speech performance. Indeed, our behavioral results revealed an enhanced lip-reading ability in CI users when compared to NH listeners (Figure 2A). Given that the two hearing groups had comparable cognitive abilities and normal or corrected-to-normal vision, this group effect may be caused by cortical reorganization as induced by auditory deprivation (*before* cochlear implantation) and/or by the limited electrical hearing with a CI (after cochlear implantation; Rouger et al., 2007; Lazard et al., 2012a). CI users may develop compensatory strategies *before* and *after* implantation to overcome the missing or limited auditory input (Layer et al., 2022; Radecke et al., 2022). Importantly, our results extend previous studies (Giraud and Truy, 2002; Rouger et al., 2007; Stropahl et al., 2015) by demonstrating that the enhanced use of visual speech cues in CI users manifests across different age groups (young and elderly). This conclusion is supported by a recent study, showing that the CI users’ increased lip-reading performance is accompanied by an enhanced recruitment of the visual cortex during audio-visual speech processing (Layer et al., 2022).

### Background-noise effect: Cochlear-implant users show a comparable noise-induced decrement in speech processing as normal-hearing listeners

The behavioral results in the EEG paradigm revealed a background-noise effect on the hit rates, with lower hit rates in the condition with background noise compared to the condition without background noise (Figure 1B). This background-noise effect on the hit rates was independent of the hearing group and of the age group (no significant interaction effects).

Similar to the behavioral results, the ERPs to sentence onset also revealed effects caused by the background noise (Figures 4A,D–F), as indicated by prolonged N1 and P2 latencies and reduced P2 amplitudes. Importantly, this background-noise effect was present regardless of the factors “age” and “hearing,” suggesting that background noise affects sensory-driven processes in speech comprehension in both the CI users and the NH listeners across different age ranges. Consistent with our results, a previous study with NH listeners revealed that noise modified the N1-P2 complex and that this noise effect was stronger for interrupted or babble noise compared to continuous noise (Papesh et al., 2015). Furthermore, the study of Finke et al. (2016a) reported prolonged N1 latencies in speech conditions with compared to without background noise, not only in NH listeners but also in CI users. These background-noise effects on sensory ERPs can be explained by the fact that speech perception in noise is more challenging than speech without any background noise. Background noise, in general, reduces the ability to understand speech because the difficulty to extract speech information in the noisy listening environment is increased on the auditory periphery and the central processing pathway (Helfer and Vargo, 2009; Romei et al., 2011).

Interestingly, a significant interaction effect between the background noise and age was found specifically for the P2 latency, as evidenced by a significantly prolonged P2 latency in the elderly individuals for the condition with compared to without background noise. By contrast, this background-noise effect on the P2 latency was not present in the younger individuals. Our results suggest that the elderly individuals have more difficulties than younger individuals in understanding speech in challenging listening conditions with a background noise. Interestingly, this background-noise effect on the P2 latency observed specifically in elderly individuals appears to be equally pronounced in the CI users and the NH listeners. Our observations are consistent with previous results from Tremblay et al. (2003), showing delayed P2 latencies in elderly individuals with and without age-related hearing loss when compared to NH younger individuals. The authors argued that aging and age-related hearing loss impair the temporal precision of response properties in the central auditory system.

The lack of an interaction between “hearing” and “condition” for sensory ERPs suggests that the chosen background-noise condition did not additionally impair the cortical speech processing in the CI users compared to the NH listeners. This lack of a group difference regarding the background-noise effect is surprising given that CI users are generally subjected to a more difficult listening condition than NH listeners due to the degraded CI input, and background noise may additionally impair speech perception in CI users. We speculate that the CI users used their good cognitive abilities (for instance working memory), which were comparable with the cognitive abilities of NH listeners, and which allowed the CI users to successfully compensate for the degraded CI input and the additional background noise. In addition, the CI users may have benefited from the moderate speed of words in our stimulus material and the resulting gaps between the words (Nakajima et al., 2000), allowing the CI users to achieve relatively high performance levels even in challenging listening conditions with background noise. Presumably, the interaction between “hearing” and “condition” could have become significant by using a more challenging speech-in-noise condition with a lower SNR and/or with a different type of background noise, for instance a modulated (Finke et al., 2016a) or an interrupted or a babble noise (Papesh et al., 2015). By contrast, the current study used a stationary ICRA noise with a low challenging SNR of 10 dB, which may explain why CI users showed no significantly stronger background-noise effect on sensory speech processing when compared to NH listeners.

In contrast to the sensory ERPs, we found no significant effects of background noise on the higher-cognitive speech processing (N400; Figures 4B,C), although the behavioral performance (in the EEG paradigm) was reduced in all participants in the condition with compared to without background noise (Figure 1B). Similar to our observations, it has been documented that increased noise levels affect sensory ERPs (P1-N1-P2) more robustly than the later ERPs reflecting higher-cognitive processing (Martin et al., 1997; Whiting et al., 1998). Nevertheless, a background-noise effect on semantic speech processing (N400) has previously been reported in NH listeners (Romei et al., 2011). We speculate that methodological differences regarding the stimulus material (sentences vs. words), the type of background noise (stationary vs. multi-talker babble) and the EEG paradigm (contextual fit vs. semantic priming) may at least partially account for the discrepancy of results between the current findings and previous observations (Romei et al., 2011). Future studies are necessary to systematically investigate the effects of different stimuli, different types of background noise and different SNRs on the semantic speech processing, in both CI users and NH listeners across different age ranges.

### Age effect: Elderly individuals show differences in cognitive abilities and speech processing compared with young individuals

We observed a significant age effect across all cognitive tests (see Figures 2B–F). Specifically, the elderly individuals showed poorer performance than younger individuals in working memory, cognitive flexibility, verbal learning and retrieval tasks. Thus, our results confirm previous reports of an age-related decline in different cognitive functions (Salthouse, 1996; Park et al., 2002; Bopp and Verhaeghen, 2005; Pliatsikas et al., 2019). However, our elderly individuals outperformed the younger ones in the verbal intelligence. These results support previous observations that not all cognitive functions are affected by the age-related decline (Glisky, 2007). Indeed, our results show that certain specific functions, in particular verbal intelligence, can even improve with age and life experience (Park et al., 2002; Kavé and Yafé, 2014; Shafto and Tyler, 2014).

One may speculate that the CI users’ cognitive performance is reduced compared to NH listeners because previous studies have shown that hearing loss is a risk factor for the development of dementia and cognitive decline (Lin et al., 2013; Thomson et al., 2017; for a review see: Wayne and Johnsrude, 2015; Fortunato et al., 2016; Livingston et al., 2020). However, studies have pointed to a positive effect of hearing rehabilitation on cognition (hearing aids: Amieva et al., 2015; Dawes et al., 2015; CI: Mosnier et al., 2015; Cosetti et al., 2016), while others have reported no or only marginal effects (Huber et al., 2021). Our results revealed no significant interaction between “age” and “hearing group,” suggesting that the age effect on cognitive functions is comparable between the CI users and the NH listeners. These findings expand previous research (Abdel-Latif and Meister, 2022) by pointing to similar cognitive abilities between CI users and NH listeners, for both elderly and young individuals. Nevertheless, future longitudinal studies are necessary to better understand the role of hearing rehabilitation on the age-related decline in cognitive abilities of CI users.

Regarding the behavioral performance in the sentence categorization task (EEG paradigm) and the clinical speech tests (speech audiometry), our results showed neither an age effect nor any interaction effects with the factor of “age”. This is in contrast to other studies reporting poorer speech intelligibility in elderly individuals compared to young ones, in particular in speech conditions with additional background noise (CI patients: Lenarz et al., 2012; NH listeners: Meister et al., 2013). The discrepancy of results may be attributable due to methodological differences between studies, particularly in terms of stimuli (words vs. meaningless sentences vs. semantically correct/incorrect sentences), task (repeat words vs. matrix test vs. semantic categorization) and participant group variability (demographic factors in CI users). In general, our finding of comparable speech performance across age groups may at least partially be explained by the moderate speed of words in our stimulus material, which allowed the elderly individuals to achieve the high performance levels of younger individuals. The individuals could benefit from the gaps between the words (Nakajima et al., 2000), which is particularly crucial in the elderly, given that brain functions slow down with age (Salthouse, 1996; Schneider et al., 2005).

In contrast to the behavioral results, we found an effect of age on the ERPs, in particular on the N1 and P2 latencies (prolonged in elderly), and the N1 amplitude (increased in elderly). These findings suggest age-related changes in the auditory periphery and/or the central auditory system in both the CI users and the NH listeners (Martin and Jerger, 2005; Humes et al., 2012). Similar to our findings, Tremblay et al. (2003) reported increased N1 and P2 latencies in elderly compared to young NH listeners, and they concluded that age affects the temporal precision in the aging central auditory system. Our results extend previous research by showing comparable age effects on sensory-driven processes in NH listeners and in CI users. The lack of a significant interaction between “age” and “hearing group” in our study suggests that the age effect on sensory speech processing is not enhanced due to the qualitatively reduced CI input.

Our results suggest that age affects not only sensory-driven processes, but also later, cognitive-linguistic integration processes, as evidenced by a reduced N400 effect in elderly compared to young individuals. Our findings confirm previous reports of an age-related decline of the N400 effect in NH listeners (Gunter et al., 1992; Iragui et al., 1996; Juottonen et al., 1996; Kutas and Iragui, 1998; Xu et al., 2017). The elderly may have a reduced N400 effect due to slower neural processing time, a slower access to the semantic memory, and a limited capacity for semantic integration (Kutas and Iragui, 1998; Schneider et al., 2005). Consistent with our findings, Faustmann et al. (2007) observed an age-related reduction in the N400 effect across different age groups of elderly NH listeners, although the N400 latency was comparable between the three elderly age groups. In our results, we did not find any age effects on the N400 latency as well. One reason for this lack may be the moderate speed of words in the sentence categorization task, which allowed the elderly individuals to keep up with the speech processing of younger adults.

We observed that elderly NH listeners and elderly CI users showed enhanced sensory-driven processing (enhanced N1 amplitude) but reduced later, cognitive-linguistic speech processing (reduced N400 effect) when compared to younger individuals. This suggests that, when compared to young individuals, elderly CI users and NH listeners require more effortful processing at early cortical stages of speech processing (enhanced N1 amplitude), which may result in fewer neural resources available for the later semantic integration processes (reduced N400 effect), given that central resources seem to be limited in capacity (Kahneman, 1973). In addition, our elderly individuals revealed reduced cognitive abilities (except the verbal intelligence) when compared to young individuals, which may further limit the capacity for cognitive-linguistic integration processes in these individuals. This assumption fits to the Ease of Language Understanding model (ELU) by Rönnberg et al. (2013), which posits that in suboptimal hearing conditions, the auditory input does not match with the stored attributes in the long-team memory (mental lexicon). These difficult listening conditions, such as speech in background noise or listening with a CI, may require an additional explicit processing (including working memory) of the incoming speech stimuli, which appears to be age-related (Rönnberg et al., 2008, 2013).

Taken together, we conclude that age-related changes in the auditory periphery and/or the central auditory system cause alterations in sensory speech processing. Elderly individuals require more neural resources for sensory speech processing (increased N1 amplitude) than young individuals. Due to these effortful sensory-driven processes, the elderly have fewer neural resources available for the higher-cognitive speech processing (reduced N400 effect), which is exacerbated by the fact that elderly individuals have reduced cognitive abilities.

### Correlation between behavioral results (electroencephalography paradigm) and clinical speech tests (speech audiometry)

Within the CI users, the behavioral performance in the sentence categorization task (with background noise) correlated with the speech intelligibility measured by the GÖSA in noise. Higher hit rates in the sentence categorization task correlated with better performances in the GÖSA in noise (smaller values of SRT reflect better speech intelligibility), indicating a connection between the different behavioral measures. Importantly, we used the same noise in the sentence categorization task as in the clinical GÖSA speech test (stationary ICRA noise). Based on the finding that the behavioral results in the EEG paradigm and the speech intelligibility assessed by the clinical GÖSA test were correlated, we suggest that our EEG paradigm could be used in the clinical context in addition to conventional speech audiometry, with the decisive advantage of having objective, electrophysiological parameters representing sensory-driven and cognitive-linguistic integration processes. In the clinical context, ERPs can be helpful for improving the evaluation of hearing rehabilitation in CI users with unclear constellation of findings, by providing an objective measure of speech processing.

## Limitations

Although we observed main effects of the different factors (“hearing group,” “background noise,” and “age effect”) on the behavioral performance and on ERPs, we did not find any interaction effects, except for a significant interaction between “background noise” and “age” for the P2 latency. Possible reasons for the lack of interaction effects could be the variability within the groups of CI users (due to the different demographic factors) and the used background noise which may not have interfered appropriately (due to the SNR being too high and/or not being individually adjusted). However, increasing the task difficulty by using lower SNRs likely leads to a reduced number of correct trials (correctly categorized sentences) for the ERP analysis, which significantly decreases the ERP data quality.

One might concern that etiology could confound our results. Indeed, previous literature has shown that etiology is a significant factor predicting the CI outcome (Goudey et al., 2021), although other predictive factors, for instance the duration of CI experience and the duration of moderate hearing loss, seem to have an even greater influence on the CI outcome (Lazard et al., 2012b). However, in our study the factor of etiology was not substantially different between young and elderly CI users (see Tables 1, 2), and thus we are confident that the factor “etiology” did not confound our results. Nevertheless, we suggest that future studies should use larger sample sizes, which allow powerful regression analyses to investigate the influence of different predicting factors, including etiology, cognitive factors, electrophysiological parameters, and the hearing status of the contralateral ear.

Currently, our ERP results are restricted to auditory conditions, and we did not investigate the interaction between our auditory ERP results and the quality-of-life of the CI users. It would be interesting to investigate the speech processing under more ecologically valid stimulus conditions, specifically in speech conditions with visual cues, and to use a quality-of-life questionnaire, which allows a more differentiated assessment of the subjective benefit and usefulness of the CI in everyday life. Indeed, our results revealed enhanced lip-reading skills in CI users than in NH listeners. By providing additional visual speech cues, we anticipate that CI users significantly improve their speech performance and ERPs in difficult listening conditions, such as speech in background noise, due to their pronounced lip-reading ability (Layer et al., 2022).

## Conclusion

The results of the current study revealed consistent age effects across CI users and NH listeners not only in the cognitive functions but also in the central auditory speech processing. In comparison to young individuals, the elderly showed *higher* verbal intelligence but *lower* performance in working memory, cognitive flexibility, verbal learning and verbal retrieval. Similarly, the CI users and the NH listeners revealed consistent age effects in cortical speech processing, suggesting that increasing age results in *prolonged* and *enhanced* perceptual processes (N1/P2), but *reduced* cognitive-linguistic integration processes (N400). Although the CI users revealed general limitations in speech conditions (behavior and ERPs), both hearing groups showed a comparable noise-induced decrement in behavioral speech performance and in cortical speech processing. In summary, these results demonstrate that cortical speech processing in CI users is impaired not only at sensory but also at higher-cognitive processing stages, both in conditions with and without background noise. Further, we conclude that elderly individuals (regardless of the hearing group), when compared to the younger ones, need more effortful processing at sensory stages of speech processing (larger N1 amplitude), which seems to be at the cost of the processing resources for cognitive-linguistic integration (smaller N400 effect). The processing resources for cognitive-linguistic integration are additionally limited by the impaired cognitive abilities in the elderly in individuals, showing for instance reduced WMC.

## Data availability statement

The raw data supporting the conclusions of this article will be made available by the authors, without undue reservation.

## Ethics statement

The studies involving human participants were reviewed and approved by the Ethics Committee of Cologne University’s Faculty of Medicine. The patients/participants provided their written informed consent to participate in this study.

## Author contributions

PB: data acquisition, data analyses, statistical analysis, writing, visualization, and project administration. VM, RL-R, and MW: writing and editing. HM: conceptualization, writing, and editing. AW: data analysis and editing. PS: conceptualization, methodology, resources, writing, supervision, project administration, funding acquisition, and data analyses. All authors contributed to the article and approved the submitted version.

## Footnotes

1
https://www.memoryclinic.ch/de/main-navigation/neuropsychologen/cerad-plus/

2
http://www.brainproducts.com
