# Supplementary material for: Age effects on cognitive functions and speech-in-noise processing: An event-related potential study with cochlear-implant users and normal-hearing listeners
Source: Front Neurosci. 2022 Dec 22;16:1005859. doi: 10.3389/fnins.2022.1005859 (PMC9815545; doi:10.3389/fnins.2022.1005859)
Supplement: Supplementary file 1 [file Data_Sheet_1.docx]

Supplementary Material

# Supplementary Data

In addition to the statistical analyses regarding the N400 amplitude of the difference waves, we performed an analysis taking the semantic context (correct/violation) of the sentences into account. Our aim was to find out if there are differences between the different groups (hearing-groups: NH/CI; age groups: young/elderly) in both semantic situations (correct/violation) and with and without background noise. Furthermore, this additional analysis was performed with a focus on four different regions-of-interest (ROIs): frontal (F: F3, Fz, F4,); frontocentral (FC: FC1, FCz, FC2), central (C: C3, Cz and C4) and centroparietal (CP: CP1, Pz, CP2).

In a previous study by Aydelott et al. (2006) with NH listeners, it was shown that the N400 effect for acoustically degraded speech condition (low-pass filtered speech at 1kHz) was decreased compared to the not degraded speech condition. Furthermore, the authors discovered in planned comparisons that in the unaltered/natural speech condition, the N400 amplitude for incongruent target words was significantly higher than for congruent targets. This significant difference was not found between the congruent and incongruent targets in the filtered context. The authors explained that this reduced N400 effect in the filtered speech condition is caused by reduced N400 amplitudes on posterior electrode sites for the incongruent targets. They assumed, that the degradation of acoustical stimuli (filtered speech condition) affects the neuronal word-processing in a context because of the reduced availability of semantic information (disrupted lexical-semantic representation) during the on-line sentence comprehension (Aydelott et al., 2006). Reduced availability of semantic context information due to the acoustical degradation may result in fewer requests for semantic integration in sentences with semantical violation, resulting in a decreased N400 effect.

The filtered speech condition in the study of Aydelott et al. (2006) could be roughly compared to the CI users (degraded CI input) in our study; thus, we suggested differences regarding the N400 amplitude in different semantic context conditions (correct/violation) between both hearing-groups (NH/CI).

**N400 Amplitude:** We computed a 2×2×2×2×4 mixed ANOVA with the factors ‘*hearing-group*’ (NH/ CI) and ‘*age group’* (young/elderly) as within-subjects factors and the factors ‘*condition*’ (with/ without background noise), ‘*semantic context*’ (correct/violation) and ‘*ROI*’ with four separate ROIs (frontal/frontocentral/central/centroparietal) as within-subjects factors for the N400 amplitude. In the case of violation of the sphericity assumption, a Greenhouse-Geisser correction was applied. Post-hoc t-tests were performed when there were significant main effects or interaction effects (p < .05), and a Holm-Bonferroni correction was used in the case of multiple comparisons.

The ANOVA showed a main effect of condition (*F*_1,48_ = 5.344*, p* = .025*, η_p_^2^* = .100), a main effect of semantic (*F*_1,48_ = 25.710*, p* = .001*, η_p_^2^* = .349) and a main effect of ROI (*F*_1.608,77.167_ = 29.041*, p* = .001*, η_p_^2^* = .377)*.* There was no main effect of hearing-group or age. In the hearing condition without noise, the N400 amplitude (regardless of age, hearing-group, semantic and ROI) was increased compared to the condition with noise (without noise mean amplitude and SD: 364.74 ± 232.81 µV × ms; with noise mean amplitude and SD: 313.18 ± 188.43 µV × ms). The N400 amplitude for semantically correct sentences was reduced compared to the semantically violated sentences (semantically correct mean amplitude and SD: 288.94 ± 193.51 µV × ms; semantically violated mean amplitude and SD: 388.97 ± 228.42 µV × ms). The main effect of ROI showed no subsequent significant difference in the comparison between the frontal and the frontocentral ROI (*t(51)* = .714, *p* = .479, *Cohen‘s d* = .099). Otherwise, all other comparisons of different ROIs were statistically significant (with Holm-Bonferroni correction). The frontal ROI shows a statistically significant difference in the N400 amplitude to the central (*t(51)* = 4.661, *p* = .001, *Cohen‘s d* = .646) and to the centroparietal ROI (*t(51)* = 5.881, *p* = .001, *Cohen‘s d* = .816). The N400 amplitude in the frontocentral ROI statistically differed from the central ROI (*t(51)* = 6.058, *p* = .001, *Cohen‘s d* = .840) and the centroparietal ROI (*t(51)* = 6.579, *p* = .001, *Cohen‘s d* = .912). Additionally, the central ROI shows a statistically different N400 amplitude compared to the centroparietal ROI (*t(51)* = .4,790, *p* = .001, *Cohen‘s d* = .664). The mean N400 amplitudes (and SD) for the different ROIs were in descending order: frontal ROI (462.56 ± 289.75 µV × ms), frontocentral ROI (442.34 ± 307.58 µV × ms), central ROI (277.38 ± 220.66 µV × ms) and centroparietal ROI (173.55 ± 164.95 µV × ms).

As it would otherwise go beyond the scope of this report, only the significant interactions are reported in the following sections. The interactions without any statistically significance are shown in the Supplementary Table 1. Interaction effects were statistically significant between the semantic context and the hearing-group (*F*_1,48_ = 7.375*, p* = .009*, η_p_^2^* = .133), between the semantic context and the factor age (*F*_1,48_ = 6.267*, p* = .016*, η_p_^2^* = .115), as well as between the semantic context, the ROI and the hearing-group (*F*_1.832,87.950_ = 4.563*, p* = .015*, η_p_^2^* = .087). All other interaction effects showed no statistical significance.

Post hoc tests were applied subsequent to the significant interaction effect between the semantic context and hearing-group. The post hoc t-tests (taking into account the Holm-Bonferroni correction) revealed a statistically significant difference for the NH listeners between the semantically correct and semantically violated condition (*t(25)* = -5.087, *p* = .001, *Cohen‘s d* = -.998) but not for the CI users (*t(25)* = -1.654, *p* = .111, *Cohen‘s d* = -.324). The N400 amplitude for the semantically correct condition was decreased compared to the violated condition for the NH listeners (semantically correct mean amplitude and SD: 249.76 ± 173.65 µV × ms; semantically violated mean amplitude and SD: 403.36 ± 251.86 µV × ms). The comparison between both hearing-groups (NH vs. CI) for both semantical sentence conditions (correct/violation) revealed no statistically significant difference (semantically correct: *t(50)* = -1.477, *p* = .146, *Cohen‘s d* = -.410, semantically violated: *t(50)* = .451, *p* = .654, *Cohen‘s d* = .125). The missing significant difference between the semantically correct and semantically violated condition in the CI user group can be explained by the reduced availability of semantic context information (especially in the sentences with semantical violation) caused by the acoustical degradation through the CI device.

Regarding the significant interaction between semantic context and age group, the post hoc tests revealed a statistically significant difference for the young individuals between the semantically correct and violated condition (*t(25)* = -6.561, *p* = .001, *Cohen‘s d* = -1.287) but not for the elderly individuals (*t(25)* = -1.452, *p* = .159, *Cohen‘s d* = -.285). The N400 amplitude for the semantically correct condition was decreased compared to the violated condition for the young individuals (semantically correct mean amplitude and SD: 246.53 ± 164.32 µV × ms; semantically violated mean amplitude and SD: 395.94 ± 204.73 µV × ms). The comparison between the age groups (young vs. elderly) for both semantical sentence conditions (correct/violation) revealed no statistically significant difference (semantically correct: *t(50)* = -1.605, *p* = .115, *Cohen‘s d* = -.445 , semantically violated: *t(50)* = .218, *p* = .828, *Cohen‘s d* = .060). The missing significant difference between the semantically correct and semantically violated condition in the elderly individuals can be explained by a slower and/or less effective processing of the context in general, resulting in a reduced N400 effect (Aydelott et al., 2006; Cameli & Phillips, n.d.; Kutas & Iragui, 1998).

The significant interaction effect between the semantic context, different ROIs and the factor hearing-group was followed up by four-way repeated measures ANOVAs (semantically correct condition and CI user, semantically violated condition and CI user, semantically correct condition and NH listeners, semantically violated condition and NH listeners). With these repeated measures ANOVAs, we focus on the comparison between the different ROIs (within subject factor: ROI) within a specific hearing-group (CI vs NH) in combination with a specific semantic context (semantical correct vs semantical violated). The N400 amplitude for the CI user group and the semantically correct condition (*F*_2.045,51.118_ = 16.092*, p* = .001*, η_p_^2^* = .392), as well as for the semantically violated condition (*F*_1.447,36.179_ = 4.572*, p* = .027*, η_p_^2^* = .155) revealed a statistically significant difference between the ROIs. Similarly, for the NH listeners the N400 amplitude revealed a statistically significant difference between the four ROIs for the semantically correct condition (*F*_1.709,42.736_ = 20.335*, p* = .001*, η_p_^2^* = .449), as well as for the semantically violated condition (*F*_1.431,35.777_ = 15.942*, p* = .001*, η_p_^2^* = .389). The significant interaction effect (semantic context × ROI × hearing group) also was followed up by post hoc tests (taking into account the Holm-Bonferroni correction) within each hearing group (CI users/NH listeners to compare the N400 amplitude between the two semantic contexts (correct vs. violated) within each ROI. Within the CI group, the post hoc analysis revealed a statistically significant difference between the correct and the violated condition in the centroparietal ROI (CP: *t(25)* = -3.361, *p* = .003, *Cohen‘s d* = -.659). The N400 amplitude in the semantically correct condition in the centroparietal ROI was reduced (mean amplitude and SD: 126.24 ± 137.87 µV×ms) compared to the semantically incorrect condition (mean amplitude and SD: 265.30 ± 265.10 µV×ms). In all other ROIs (F, FC, C) no statistically significant difference in N400 amplitude was observed between both context conditions. Within the NH listener group, all ROIs revealed a statistically significant difference between both semantic conditions (F:  *t(25)* = -3.847, *p* = .001, *Cohen‘s d* = -.754; FC: *t(25)* = -3.917, *p* = .001, *Cohen‘s d* = -.768; C:  *t(25)* = -4.570, *p* = .001, *Cohen‘s d* = -.896; CP: *t(25)* = -3.847, *p* = .001, *Cohen‘s d* = -.754). The N400 amplitude was reduced in the semantically correct condition when compared with the semantically violated condition in all ROIs (for mean and SD see Table2b). The post hoc tests to compare the hearing groups (CI vs. NH) within each ROI (F, FC, C, CP) for each semantic condition (correct, violated) revealed no statistically significant differences.

All results of the different post hoc t-tests (with Holm-Bonferroni correction) for each repeated measures ANOVA are listed in the supplementary Table 2a&b as well as the corresponding mean values and standard deviations of the N400 amplitude.

Summing up the main results of the supplementary analyses showed, that regarding the semantic context (correct vs. violation) there was a statistically significant difference of the N400 amplitude among the NH listeners, but not among the CI users (post hoc tests regarding the interaction semantic context × hearing-group). For the NH listeners, the N400 amplitude was decreased in the semantically correct condition compared to the violated condition, whereas the N400 amplitude was similar for both semantic context situations in CI users. Although there was no direct statistically significant difference between NH listeners and CI users, a significant difference between the semantic conditions in the CI users was missing, (contrary to the NH listeners), which shows a difference, between the hearing-groups, at least indirectly.

The post hoc analyses regarding the significant interaction of semantic context × ROI × hearing-group revealed a statistical difference in all ROIs (F, FC, C, CP) between the two semantic context conditions (correct vs. violation) for the NH listeners. For the CI users, a statistical difference of the N400 amplitude between the correct and violated condition was only found in the centroparietal ROI, but not in the other ROIs (F, FC, C). When comparing the two hearing-groups (CI users vs NH listeners), there were no differences in the respective semantic context conditions (correct, violated) per ROI (F, FC, C, CP).

The interaction effect semantic context and age was statistically significant, and the subsequent post hoc t-tests revealed a significant difference in the younger individuals between the N400 amplitude in the correct and violated semantic context independently of the ROI. The N400 amplitude was reduced for the correct semantic context in comparison to the violated context. This significant difference was not observed in the elderly individuals. There was no interaction between semantic context, ROI and age group, thereby no post-hoc tests were conducted.

We did not only look at the difference wave (N400) and analysed it statistically, but also considered the ERP wave curves regarding the semantic context. The statistical analysis of these ERP wave curves in relation to the semantic context is intriguing in order to uncover differences on a semantic level across different age and hearing-groups. The statistically significant difference of N400 amplitude between the correct and violated semantic context was observable for the NH listeners (but not for the CI users) and for the younger individuals (but not for the elderly individuals). Therefore, one can assume that the degraded CI signal and also the factor age alter the sentence processing with respect to the semantic context. The N400 amplitude was reduced in the semantically violated sentences for the CI users and the elderly individuals, respectively, resulting in a reduced N400 effect in these groups.

# References

Aydelott, J., Dick, F., & Mills, D. L. (2006). Effects of acoustic distortion and semantic context on event-related potentials to spoken words. *Psychophysiology, 43*(5), 454–464. https://doi.org/10.1111/j.1469-8986.2006.00448.x

Cameli, L., & Phillips, N. A. (n.d.). Age-related differences in semantic priming: evidence from event-related brain potentials. *Brain and Cognition, 43*(1–3), 69–73. http://www.ncbi.nlm.nih.gov/pubmed/10857666

Kutas, M., & Iragui, V. (1998). The N400 in a semantic categorization task across 6 decades. *Electroencephalography and Clinical Neurophysiology/Evoked Potentials Section, 108*(5), 456–471. https://doi.org/10.1016/S0168-5597(98)00023-9

# Supplementary Figures and Tables

## Supplementary Tables

**Supplementary Table1.** Results of mixed 2×2×2×2×4 ANOVA. Bold: statistically significant.

| **Effect** | **Result** |
| --- | --- |
| **Condition** | ***F*_1,48_ = 5.344*, p* = .025*, η_p_^2^* = .100** |
| Condition × hearing-group | *F*_1,48_ = .174*, p* = .678*, η_p_^2^* = .004 |
| Condition × age | *F*_1,48_ = .235*, p* = .630*, η_p_^2^* = .005 |
| Condition × hearing-group × age | *F*_1,48_ = .427*, p* = .517*, η_p_^2^* = .009 |
| **Semantic** | ***F*_1,48_ = 25.710*, p* = .001*, η_p_^2^* = .349** |
| **Semantic × hearing-group** | ***F*_1,48_ = 7,375*, p* = .009*, η_p_^2^* = .133** |
| **Semantic × age** | ***F*_1,48_ = 6,267*, p* = .016*, η_p_^2^* = .115** |
| Semantic × hearing-group x age | *F*_1,48_ = ,360*, p* = .551*, η_p_^2^* = .007 |
| **ROI** | ***F*_1.608,77.167_ = 29.041*, p* = .001*, η_p_^2^* = .377** |
| ROI × hearing-group | *F*_1.608,77.167_ = .162*, p* = .804*, η_p_^2^* = .003 |
| ROI × age | *F*_1.608,77.167_ = 1.320*, p* = .269*, η_p_^2^* = .027 |
| ROI × hearing-group × age | *F*_1.608,77.167_ = .292*, p* = .699*, η_p_^2^* = .006 |
| Condition × semantic | *F*_1,48_ = .501*, p* = .483*, η_p_^2^* = .010 |
| Condition × semantic × hearing-group | *F*_1,48_ = .991*, p* = .324*, η_p_^2^* = .020 |
| Condition × semantic × age | *F*_1,48_ = .572*, p* = .453*, η_p_^2^* = .012 |
| Condition × semantic × hearing-group × age | *F*_1,48_ = .241*, p* = .626*, η_p_^2^* = .005 |
| Condition × ROI | *F*_1.794,86.125_ = 580*, p* = .544*, η_p_^2^* = .012 |
| Condition × ROI × hearing-group | *F*_1.794,86.125_ = .074*, p* = .912*, η_p_^2^* = .002 |
| Condition × ROI × age | *F*_1.794,86.125_ = .772*, p* = .452*, η_p_^2^* = .016 |
| Condition × ROI × hearing-group × age | *F*_1.794,86.125_ = .292*, p* = .724*, η_p_^2^* = .006 |
| Semantic × ROI | *F*_1.832,87.950_ = .322*, p* = .706*, η_p_^2^* = .007 |
| **Semantic × ROI × hearing-group** | ***F*_1.832,87.950_** **= 4.563*, p* = .015*, η_p_^2^* = .087** |
| Semantic × ROI × age | *F*_1.832,87.950_ = 2.682*, p* = .079*, η_p_^2^* = .053 |
| Semantic × ROI × hearing-group × age | *F*_1.832,87.950_ = .772*, p* = .455*, η_p_^2^* = .016 |
| Condition × semantic × ROI | *F*_1.631,78.283_ = .764*, p* = .445*, η_p_^2^* = .016 |
| Condition × semantic × ROI × hearing-group | *F*_1.631,78.283_ = .755*, p* = .448*, η_p_^2^* = .015 |
| Condition × semantic × ROI × age | *F*_1.631,78.283_ = .131*, p* = .836*, η_p_^2^* = .003 |
| Condition × semantic x ROI × hearing-group × age | *F*_1.631,78.283_ = .790*, p* = .434*, η_p_^2^* = .016 |
| Hearing-group | *F*_1,48_ = .199*, p* = .657*, η_p_^2^* = .004 |
| Age | *F*_1,48_ = .408*, p* = .526*, η_p_^2^* = .008 |
| Hearing × age | *F*_1,48_ = .578*, p* = .451*, η_p_^2^* = .012 |

**Supplementary Table2a.** Results of subsequent post hoc t-tests of the statistically significant repeated measured ANOVA. Bold: statistically significant with respect of Holm-Bonferroni correction. F: Frontal, FC: Frontocentral, C: Central, CP: Centroparietal.

| **ANOVA** | **Post-hoc t-tests** | **N400 amplitude mean and SD** |
| --- | --- | --- |
| CI user group and the semantically correct condition:  *F*_2.045,51.118_ = 16.092*, p* = .001*, η_p_^2^* = .392 | F vs. FC: *t(25)* = 1.135, *p* = .267, *Cohen‘s d* = .223 **F vs. C: *t(25)* = 3.385, *p* = .002, *Cohen‘s d* = .664 F vs. CP: *t(25)* = 4.886, *p* = .001, *Cohen‘s d* = .958 FC vs. C: *t(25)* = 3.351, *p* = .003, *Cohen‘s d* = .657 FC vs. CP: *t(25)* = 4.930, *p* = .001, *Cohen‘s d* = .967 C vs CP: *t(25)* = 3.812, *p* = .001, *Cohen‘s d* = .748** | F: 488.60 ± 324.09 µV×ms  FC: 428.90 ± 338.18 µV×ms  C: 268.78 ± 251.66 µV×ms  CP: 126.24 ± 137.87 µV×ms |
| CI user group and the semantically violated condition  *F*_1.447,36.179_ = 4.572*, p* = .027*, η_p_^2^* = .155 | F vs. FC: *t(25)* = .333, *p* = .742, *Cohen‘s d* = .065 F vs. C: *t(25)* = 1.950, *p* = .062, *Cohen‘s d* = .382 F vs. CP: *t(25)* = 2.099, *p* = .046, *Cohen‘s d* = .412 **FC vs. C: *t(25)* = 3.036, *p* = .006, *Cohen‘s d* = .595** **FC vs. CP: *t(25)* = 2.786, *p* = .010, *Cohen‘s d* = .546** C vs CP: *t(25)* = 1.151, *p* = .261, *Cohen‘s d* = .226 | F: 474.94 ± 340.92 µV×ms  FC: 455.35 ± 316.55 µV×ms  C: 302.73 ± 274.31 µV×ms  CP: 265.30 ± 265.10 µV×ms |
| NH listener group and the semantically correct condition  *F*_1.709,42.736_ = 20.335*, p* = .001*, η_p_^2^* = .449 | F vs. FC: *t(25)* = .037, *p* = .971, *Cohen‘s d* = .007 **F vs. C: *t(25)* = 3.836, *p* = .001, *Cohen‘s d* = .752 F vs. CP: *t(25)* = 4.875, *p* = .001, *Cohen‘s d* = .956 FC vs. C: *t(25)* = 4.617, *p* = .001, *Cohen‘s d* = .905 FC vs. CP: *t(25)* = 5.387, *p* = .001, *Cohen‘s d* = 1.056  C vs CP: *t(25)* = 4.198, *p* = .001, *Cohen‘s d* = .823** | F: 348.64 ± 251.32 µV×ms  FC: 347.57 ± 271.86 µV×ms  C: 198.27 ± 152.86 µV×ms  CP: 104.57 ± 123.52 µV×ms |
| NH listener group and the semantically violated condition  *F*_1.431,35.777_ = 15.942*, p* = .001*, η_p_^2^* = .389 | F vs. FC: *t(25)* = .016, *p* = .988, *Cohen‘s d* = .003 **F vs. C: *t(25)* = 3.993, *p* = .001, *Cohen‘s d* = .783 F vs. CP: *t(25)* = 4.108, *p* = .001, *Cohen‘s d* = .806 FC vs. C: *t(25)* = 4.559, *p* = .001, *Cohen‘s d* = .894 FC vs. CP: *t(25)* = 4.318, *p* = .001, *Cohen‘s d* = .847 C vs CP: *t(25)* = 3.177, *p* = .004, *Cohen‘s d* = .623** | F: 538.08 ± 361.86 µV×ms  FC: 537.52 ± 401.95 µV×ms  C: 339.76 ± 247.28 µV×ms  CP: 198.09 ± 185.36 µV×ms |

**Supplementary Table2b.** Results of subsequent post hoc t-tests of the statistically significant repeated measured ANOVA. Bold: statistically significant with respect of Holm-Bonferroni correction. F: Frontal, FC: Frontocentral, C: Central, CP: Centroparietal.

| **Group** | **Post-hoc t-tests** | **N400 amplitude mean and SD** |
| --- | --- | --- |
| CI user group | semantically correct vs. semantically violated: F: *t(25)* = .238 , *p* = .814, *Cohen‘s d* = .047 FC: *t(25)* = -.521 , *p* = .607, *Cohen‘s d* = -.102 C: *t(25)* = -1.106 , *p* = .279, *Cohen‘s d* = -.217 **CP: *t(25)* = -3.361, *p* = .003, *Cohen‘s d* = -.659** | Semantically correct: F : 488.60 ± 324.09 µV×ms FC: 428.90 ± 338.18 µV×ms C: 268.78 ± 251.66 µV×ms CP: 126.24 ± 137.87 µV×ms  Semantically violated: F: 474.94 ± 340.92 µV×ms FC: 455.35 ± 316.55 µV×ms C: 302.73 ± 274.31 µV×ms CP: 265.30 ± 265.10 µV×ms |
| NH listener group | semantically correct vs. semantically violated: **F: *t(25)* = -3.847, *p* = .001, *Cohen‘s d* = -.754 FC: *t(25)* = -3.917, *p* = .001, *Cohen‘s d* = -.768 C: *t(25)* = -4.570, *p* = .001, *Cohen‘s d* = -.896 CP: *t(25)* = -3.847, *p* = .001, *Cohen‘s d* = -.754** | Semantically correct: F: 348.64 ± 251.32 µV×ms FC: 347.57 ± 271.86 µV×ms C: 198.27 ± 152.86 µV×ms CP: 104.57 ± 123.52 µV×ms  Semantically violated: F: 538.08 ± 361.86 µV×ms FC: 537.52 ± 401.95 µV×ms C: 339.76 ± 247.28 µV×ms CP: 198.09 ± 185.36 µV×ms |
| CI user group vs NH listener group | Semantically correct: F: *t(50)* = -1.740, *p* = .088, *Cohen‘s d* = -.483 FC: *t(50)* = -.956, *p* = .344, *Cohen‘s d* = -.265 C: *t(41.236)* = -1.221, *p* = .229, *Cohen‘s d* = -.339 CP: *t(50)* = -.597, *p* = .553, *Cohen‘s d* = -.166  Semantically violated: F: *t(50)* = .648, *p* = .520, *Cohen‘s d* = .180 FC: *t(50)* = .819, *p* = .417, *Cohen‘s d* = .227 C: *t(50)* = .511, *p* = .611, *Cohen‘s d* = .142 CP: *t(50)* = .-1.059, *p* = .294, *Cohen‘s d* = -.294 | See above |

## Figures


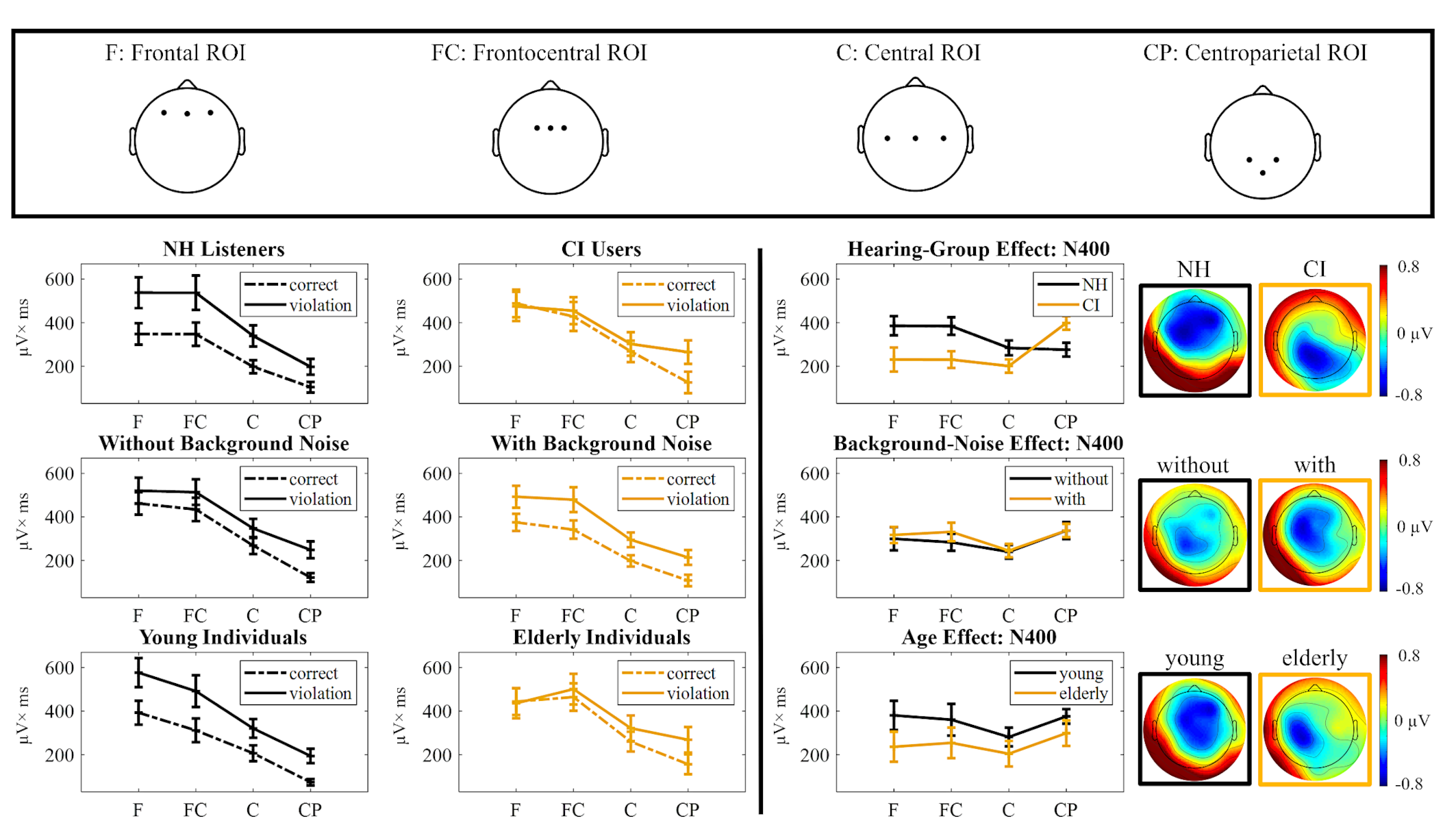


**Supplementary Fig1.** The comparison of the N400 mean amplitude in the different semantic context situations (correct, violation) separated regarding the hearing-groups (NH listeners vs. CI users), the background effect (without vs. with background noise) and the age groups (young vs. elderly individuals) are shown in the left columns. In the right column, the N400 amplitudes are plotted according to the corresponding effects (hearing-group, background-noise, age effect) and the maps with the topographical distribution are shown. The significant differences after the post hoc tests are not marked here for the sake of clarity but are shown in the tables.


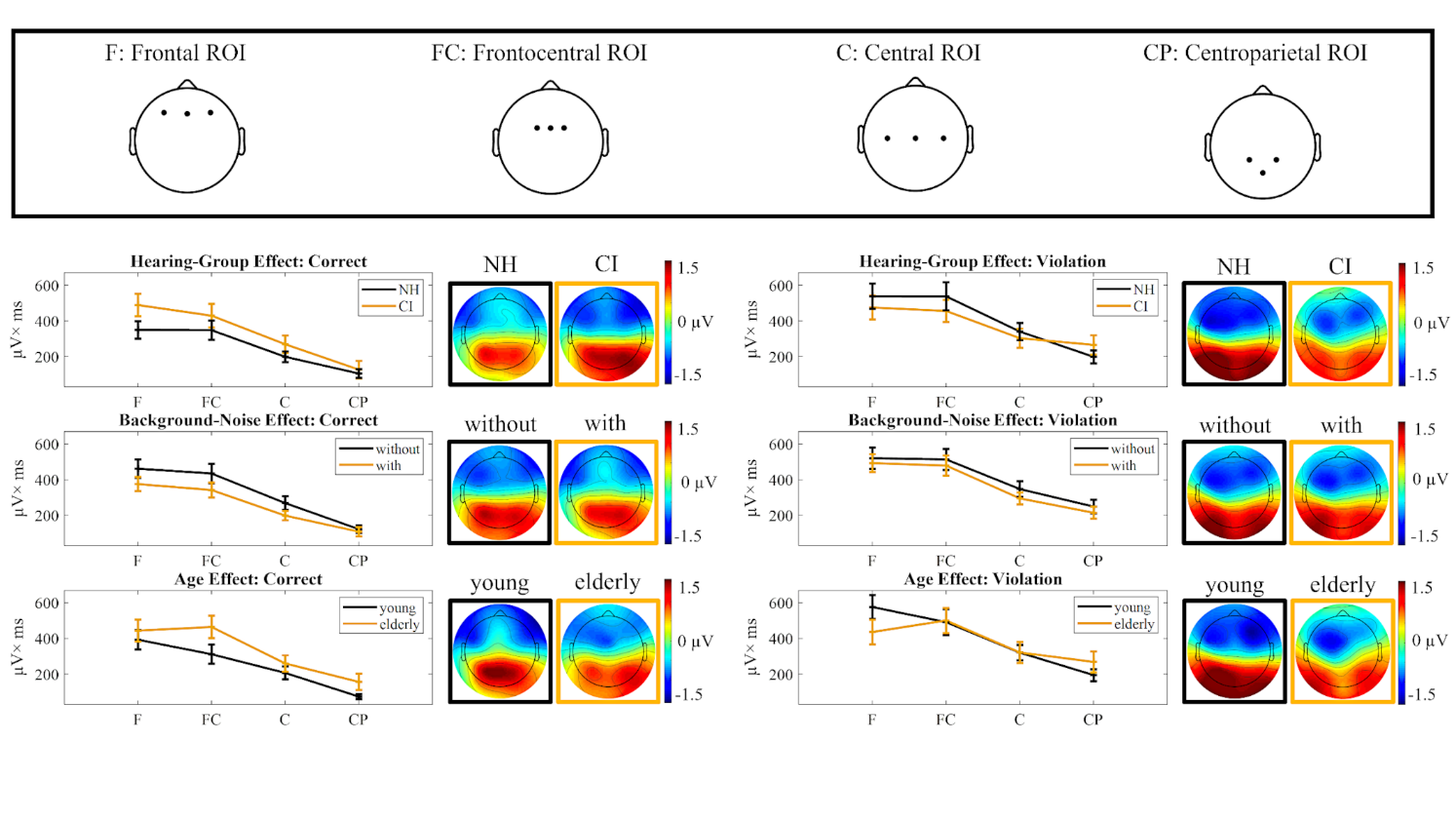


**Supplementary Fig2.** The comparison of the N400 mean amplitude between the hearing-groups (NH vs CI), between the background-noise conditions (without vs with background-noise) and between the age groups (young vs. elderly) are separately shown for the semantic correct context situation (left column) and for the semantic violated context situation (right column). The maps with the topographical distribution are also depicted. The significant differences after the post hoc tests are not marked here for the sake of clarity but are shown in the tables.
